# Engineered nanoparticles bind elapid snake venom toxins and inhibit venom-induced dermonecrosis

**DOI:** 10.1371/journal.pntd.0006736

**Published:** 2018-10-04

**Authors:** Jeffrey O’Brien, Shih-Hui Lee, José María Gutiérrez, Kenneth J. Shea

**Affiliations:** 1 Department of Chemistry, University of California, Irvine, Irvine, California, United States of America; 2 Instituto Clodomiro Picado, Facultad de Microbiología, Universidad de Costa Rica, San José, Costa Rica; Instituto Butantan, BRAZIL

## Abstract

Envenomings by snakebites constitute a serious and challenging global health issue. The mainstay in the therapy of snakebite envenomings is the parenteral administration of animal-derived antivenoms. Significantly, antivenoms are only partially effective in the control of local tissue damage. A novel approach to mitigate the progression of local tissue damage that could complement the antivenom therapy of envenomings is proposed. We describe an abiotic hydrogel nanoparticle engineered to bind to and modulate the activity of a diverse array of PLA_2_ and 3FTX isoforms found in Elapidae snake venoms. These two families of protein toxins share features that are associated with their common (membrane) targets, allowing for nanoparticle sequestration by a mechanism that differs from immunological (epitope) selection. The nanoparticles are non-toxic in mice and inhibit dose-dependently the dermonecrotic activity of *Naja nigricollis* venom.

## Author summary

Snake envenoming affects approximately 2.5 million people and causes more than 100,000 deaths annually. The WHO includes envenoming as a category A Neglected Tropical Disease. Therapy involves intravenous delivery of animal-derived antivenoms, constituted by IgG from the plasma of mammals immunized with venom. Antivenoms are species-specific, expensive and suffer the limitations of a biological therapy. There are no broad-spectrum antivenoms. The work describes hope for treatment of snakebite, a broad-spectrum antivenom comprised polymer nanoparticles (NPs) engineered to sequester the major protein toxins in elapid snakes. The stable, low-cost NPs can be administered subcutaneously immediately after the bite at the site of envenoming to halt or reduce the extent of local tissue damage and mitigate the systemic distribution of toxins post-envenoming.

## Introduction

Envenomings by snakebites constitute a serious and challenging global health issue, which affects approximately 2.5 million people and causes more than 100,000 deaths annually. This situation is particularly acute in impoverished rural settings of sub-Saharan Africa, Asia and Latin America [1–4]. In addition, an estimated number of 400,000 people suffering snakebite envenomings end up with permanent physical and psychological sequelae which greatly affect their quality of life and generates a wave of social suffering in their families and communities [4, 5]. Owing to its worldwide impact, the World Health Organization (WHO) recently included snakebite envenoming as a category A disease in its list of Neglected Tropical Diseases [4]. The mainstay in the therapy of snakebite envenomings is the parenteral administration of animal-derived antivenoms, constituted by IgG or IgG fragments purified from the plasma of large animals immunized with venoms [6, 7]. When prepared by using appropriate mixtures of venoms for immunization and following Good Manufacturing Practices (GMPs), antivenoms are safe and effective drugs which, if administered timely, can control the main pathophysiological manifestations of envenomings, especially those associated with systemic effects [6, 7].

However, there are several issues related to antivenom therapy that compromise their effectiveness. These include, but are not limited to, the fact that antivenoms must be administered in a timely manner in health facilities by trained health staff, thus limiting their use in rural settings in countries where public health services and health personnel are scarce [8]. Effective treatment is further complicated by the fact that antivenoms are specific for venoms used in immunization and those of closely related snake species [7]. Significantly, antivenoms are only partially effective in the control of local tissue damage characteristic of most viperid and some elapid snakebite envenomings, mostly owing to the rapid development of these effects and the often-delayed administration of antivenoms [9, 10]. These delays can lead to permanent tissue damage and sequelae of various sorts [11–14]. There is a need therefore to develop novel therapeutic interventions, which could be administered in the field immediately following envenoming to arrest or mitigate the progression of local tissue damage and would hence complement the antivenom therapy of envenomings. Solutions to this problem must grapple with the biological complexity of snake venom as well as the social and economic challenges associated with the production, distribution, storage and timely administration of the therapy. An ideal fast response therapy should be administered safely and effectively in a rural setting and be capable of inhibiting venom from diverse venomous species.

From a molecular standpoint, antivenom functions through a concerted neutralization process involving the interaction between polyclonal antibodies and their toxin-targets. Owing to the specificity of antibodies, the complexity of venoms requires an equally complex antibody mixture capable of selectively interacting with and neutralizing relevant venom proteins. As a consequence, toxins which are not recognized by antibodies are not neutralized. To maintain bioactivity with increasingly diverse chemical composition, venomous animals have hijacked along evolution privileged protein scaffolds with high densities of conserved disulfide cross-links capable of withstanding diversification of amino-acid compositions on the surface [15]. A process of accelerated evolution of snake venom toxins has resulted in the generation of great variation in surface residues which confer a wide spectrum of toxic activities within a limited number of molecular scaffolds [16, 17]. This has an obvious impact in the ability of antibodies to recognize variable epitopes in toxins. This balance between diversity and homology is clearly manifest in snakes from the Elapidae family where most toxins consist of either the phospholipase A_2_ (PLA_2_, ~14 kDa) or the three finger toxin (3FTX, ~8 kDa) scaffold [4].

**Fig 1:**
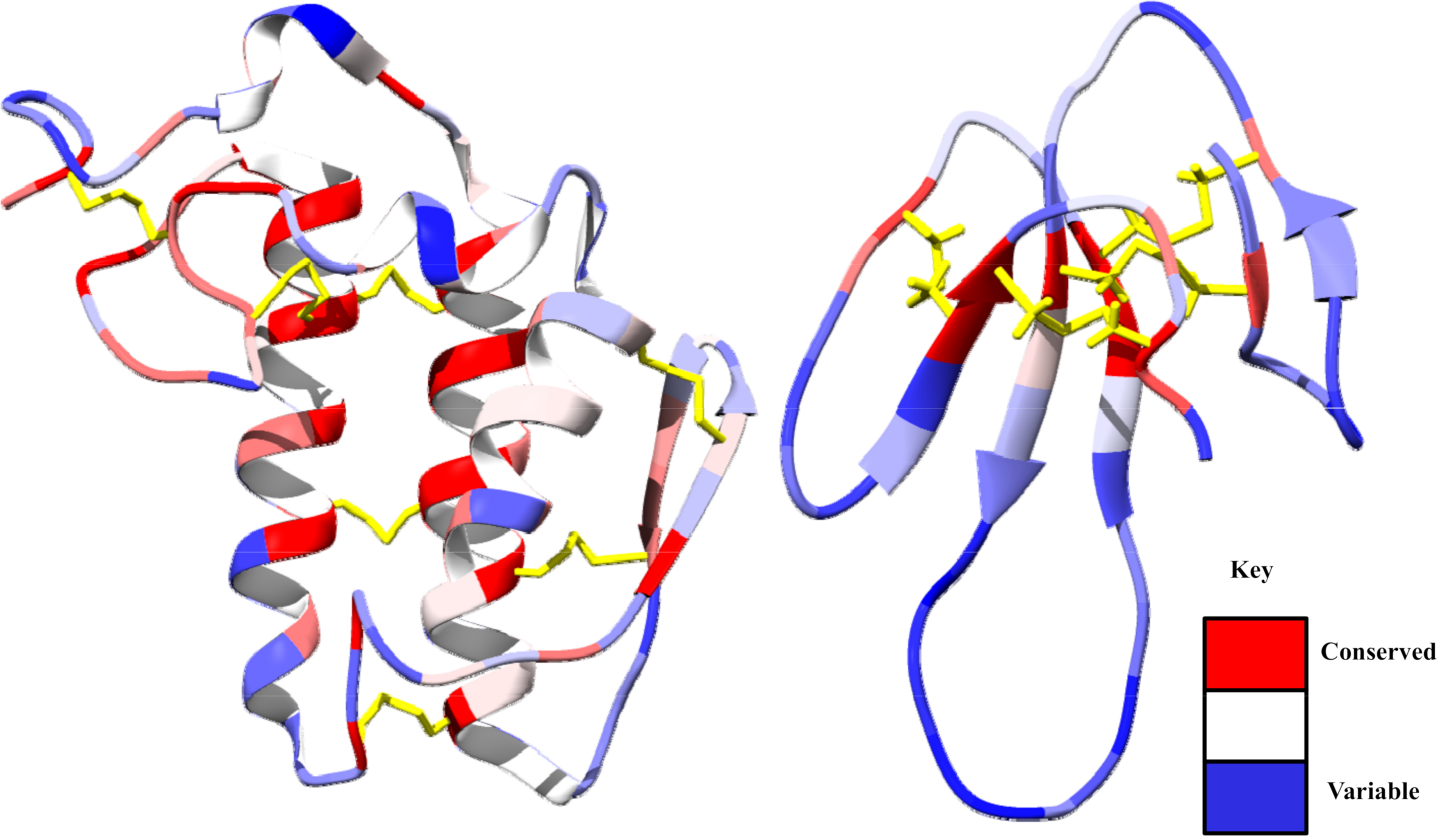
**Regions of conserved (red) and variable (blue) amino acids from reviewed sequences on www.uniprot.org for PLA**_**2**_ **(left, 27 sequences) and 3FTXs (right, 96 sequences) from the following elapid snake venoms:** *Naja sputatrix*, *Naja mossambica*, *Bungarus caeruleus*, *Bungarus fasciatus*, *Naja haje*, *Naja melanoleuca*, *Naja nivea*, *Dendroaspis polylepsis*. The core structures depicted for PLA_2_ and 3FTXs are from *Bungarus caeruleus* (PDB: 2OSN) and *Dendroaspis polylepsis* (PDB: 2MFA), respectively. Disulfide bonds are highlighted in yellow.

Both PLA_2_ and 3FTX protein families have many hundreds of isoforms capable of producing a range of pathophysiological effects [18]. However, amidst this immunological and functional variability, many share similar functional features that are distinct from abundant endogenous human proteins (Fig 1). Both toxin families have members that bind to cellular membranes and either catalyze the hydrolysis of phospholipids and hence the degradation of membrane bilayers (PLA_2_) or imbed themselves into membranes causing membrane disorganization and cytotoxicity (cytotoxic 3FTXs) [19, 20]. Other 3FTXs bind with high affinity to receptors in muscle cells or neurons, thus exerting a neurotoxic effect [21]. PLA_2_ isoforms, which range from acidic to basic, generally maintain a conserved catalytic domain, which entails a hydrophobic cavity capable of extruding glycerophospholipids from phospholipid bilayers or lipoprotein particles [22, 23]. Similarly, 3FTXs are generally cationic at neutral pH, however they have varying degrees of hydrophobicity in the loop regions that dictate the ultimate target of the toxin [24, 25]. These exposed loop regions from both protein families contain the greatest variability (Fig 1).

The chemical and structural diversity of PLA_2_ and 3FTX toxins are sufficient to challenge development of a broad-spectrum antibody preparation. However, these two families of protein toxins share features that are associated with their common targets allowing for sequestration by a mechanism that differs from immunological selection. The NPs described in this report “recognize” common structural features and broader physical properties of these toxins and may mimic their biological target (cell membranes). This “lower resolution” affinity provides an opportunity for a wide coverage of recognition/neutralization, in contrast to antibodies. Herein, we describe an abiotic hydrogel nanoparticle that indiscriminately associates with and modulates the activity of a diverse array of PLA_2_ and 3FTX isoforms.

## Methods and materials

### Materials

The following materials were obtained from commercial sources: *N*-isopropylacrylamide (NIPAm), ammonium persulfate (APS), *N*-phenylacrylamide, acrylic acid, sodium dodecyl sulfate (SDS) was obtained from Aldrich Chemical Company, Inc.; *N*,*N’*-methylenebisacrylamide (Bis) was from Fluka. All other solvents and chemicals were obtained from Fisher Scientific Inc. or VWR International LLC. NIPAm was recrystallized from hexanes before use. Water used in polymerization and characterization was purified using a Barnstead Nanopure Diamond system. 12–14 kDa MWCO cellulose membranes were purchased from Spectrum Laboratories. Precast SDS-PAGE gels (4–15% Mini-Protean), Coomassie Brilliant Blue R-250 and molecular weight ladder (Precision plus protein standards) were purchased from Bio-rad Laboratories. Human serum was purchased from Lampire Biological Laboratories. *Bungarus caeruleus* and *Naja sputatrix* venom was purchased from Sigma Aldrich. *Naja nigricollis* venom was purchased from Latoxan. *Naja mossambica*, *Bungarus fasciatus*, *Naja haje*, *Naja nivea*, *Naja melonoleuca* and *Dendroaspis polylepsis* venom was obtained from LUNA Innovations Inc. All other materials were purchased from commercial sources.

### Instrumentation

Nanoparticle size and polydispersity was determined using a Malvern ZEN3600 dynamic light scattering (DLS) instrument with a disposable sizing cuvette. Lyophilization of polymer samples was performed using a Labconco Freezone 4.5. TEM image was obtained on a FEI Tecnai G2 TF20 high resolution TEM operated with an accelerating voltage of 200 kV.

### Nanoparticle synthesis

*N*-phenylacrylamide (191.33 mg), *N*-isopropylacrylamide (191.33 mg), acrylic acid (44.57 mg) *N*, *N’-*methylenebisacrylamide (75.15 mg) and sodium dodecylsulfate (30 mg, 1 μmol) were dissolved in a 10% acetone solution in water (50 mL) to a final monomer concentration of 65 mM. The resulting solution was degassed with nitrogen for 30 min while stirring. Ammonium persulfate (30 mg dissolved in 1 mL H_2_O) was added to the degassed solution, and the reaction mixture was heated to 60°C under nitrogen for 3 h. The polymerization was quenched by exposing the reaction mixture to air, and the reaction mixture was transferred to a 12,000–14,000 MWCO membrane and dialyzed against an excess of deionized water (changed twice a day) for 4 d. 72% yield.

### Nanoparticle characterization

Dynamic light scattering (DLS) was used to characterize the size and dispersity of the nanoparticle after dialysis. Nanoparticle measurements were performed at 37°C in PBS (Dulbecco’s) and allowed to equilibrate at the designated temperature for 200 seconds prior to each measurement and were measured at a scattering angle of 173°. Concentration and yield was determined by lyophilizing a known volume of the purified nanoparticle suspension and weighing the obtained dry polymer samples.

### TEM characterization

8 μL of nanoparticles (1 mg/mL) was injected on a glow discharged TEM grid (Ultrathin Carbon Type-A, 400 mesh) and let stand for 1 min. The solution was removed by filter paper and the grid was left to air dry for 10 min. Next the grid was loaded into the instrument (FEI Tecnai G2 TF20 high resolution TEM operated at an accelerating voltage of 200 kV) for imaging.

### Venom selectivity in human serum

In a microcentrifuge tube, the nanoparticle (1 mg/mL final concentration) was incubated with human serum (25% final concentration) in phosphate buffer saline (Dulbecco’s) for 5 min at 37°C. Next, venom (250 μg/mL final concentration) was added to the nanoparticle/human serum mixture and incubated for an additional 45 min at 37°C. The suspension was then centrifuged at 10,000 RPM for 10 min, and the supernatant was replaced with fresh phosphate buffer saline four consecutive times or until the supernatant was depleted of protein by SDS-PAGE (commassie blue stain). Next, 20 μL of SDS-PAGE sample preparation mixture was added to the nanoparticle pellet, mixed and heated to 95°C for 10 minutes. Finally, the mixture was centrifuged at 5,000 RPM and the NP desorbed proteins were analyzed by SDS-PAGE (100 V).

### In gel digest

The designated slices were cut to 1mm by 1 mm by 1 mm cubes and destained 3 times by first washing with 100 μL of 100 mM ammonium bicarbonate for 15 min, followed by addition of the same volume of acetonitrile (ACN) for 15 min. The supernatant was collected and samples were dried in a speedvac. Samples were then reduced by mixing with 200 μL of 100 mM ammonium bicarbonate-10 mM DTT and incubated at 56°C for 30 minutes. The liquid was removed and 200 μL of 100 mM ammonium bicarbonate-55mM iodoacetamide was added to gel pieces and incubated at room temperature in the dark for 20 min. After the removal of the supernatant and one wash with 100 mM ammonium bicarbonate for 15 min, the same volume of ACN was added to dehydrate the gel pieces. The solution was then removed and samples were dried in a speedvac. For digestion, a sufficient solution of ice-cold trypsin (0.01 μg/μL) in 50 mM ammonium bicarbonate was added to cover the gel pieces which were set on ice for 30 min. After complete rehydration, the excess trypsin solution was removed, replaced with fresh 50 mM ammonium bicarbonate, and left overnight at 37°C. The peptides were extracted twice by the addition of 50 μL of 0.2% formic acid and 5% ACN followed by vortex mixing at room temperature for 30 min. The supernatant was removed and saved. A total of 50 μL of 50% ACN-0.2% formic acid was added to the sample, which was vortexed again at room temperature for 30 min. The supernatant was removed and combined with the supernatant from the first extraction. The combined extracts were analyzed directly by liquid chromatography (LC) in combination with tandem mass spectroscopy (MS/MS) using electrospray ionization.

### LC-MS/MS analysis

LC-MS-MS: Trypsin-digested peptides were analyzed by ultra high pressure liquid chromatography (UPLC) coupled with tandem mass spectrometry (LC-MS/MS) using nano-spray ionization. The nanospray ionization experiments were performed using a Orbitrap fusion Lumos hybrid mass spectrometer (ABSCIEX) interfaced with nano-scale reversed-phase UPLC (Thermo Dionex UltiMate 3000 RSLC nano System) using a 25 cm, 75-micron ID glass capillary packed with 1.7-μm C18 (130) BEHTM beads (Waters corporation). Peptides were eluted from the C18 column into the mass spectrometer using a linear gradient (5–80%) of ACN (Acetonitrile) at a flow rate of 375 μL/min for 1h. The buffers used to create the ACN gradient were: Buffer A (98% H_2_O, 2% ACN, 0.1% formic acid) and Buffer B (100% ACN, 0.1% formic acid). Mass spectrometer parameters are as follows; an MS1 survey scan using the orbitrap detector (mass range (m/z): 400–1500 (using quadrupole isolation), 120000 resolution setting, spray voltage of 2200 V, Ion transfer tube temperature of 275°C, AGC target of 400000, and maximum injection time of 50 ms) was followed by data dependent scans (top speed for most intense ions, with charge state set to only include +2–5 ions, and 5 second exclusion time, while selecting ions with minimal intensities of 50000 in which the collision event was carried out in the high energy collision cell (HCD Collision Energy of 30%), and the fragment masses were analyzed in the ion trap mass analyzer (With ion trap scan rate of turbo, first mass m/z was 100, AGC Target 5000 and maximum injection time of 35ms). Data analysis was carried out using the Byonic (Protein Metrics Inc.). The excel files for each set of experiments are included in the Supporting information (S1 File).

### In vitro *Naja nigricollis* and *Naja mossambica* venom inhibition

The rat skeletal muscle myoblast cell line, L6, was offered by a collaborator, Luna. L6 cells were grown in 150 cm^2^ flasks in culture DMEM media supplemented with 10%FBS and 1% penicillin, incubated at 37°C with 5% CO_2_. Cells were lifted using the trypsin and centrifuged at 800 rpm for 5 min to obtain the pellet. The cell pellet was suspended in culture DMEM media supplemented with 2% FBS and 1% penicillin and seeded into 96 well-well cell culture plates (about 1,500 cells /per well counted by cell counter). Plates were incubated at 37°C with 5% CO_2_. Media changed from the wells every second day until cell differentiation (appearance of long striated cells, observed by eye using light microscope) was observed.

### Cell proliferation assay

Concentration dependence of *Naja nigricollis* venom. Media were removed from the wells and wells were washed once with pre-warmed PBS. *Naja nigricollis* venom stock solution was diluted to 1 to 30 μg/mL in DMEM media and added into the wells. After 5min, 80 μg/mL nanoparticle was added into the wells. Culture media controls (cell and media with no venom) and the control without the addition of the nanoparticles were also run in parallel. The plate was incubated at 37°C with 5% CO_2_ for 14h. After 14h, the cell culture plate was removed from the incubator and washed with pre-warmed PBS three times. Fresh DMEM media (50 mL) and MTT (3-(4,5-dimethylthiazol-2-yl)-2,5-diphenyltetrazolium bromide from Molecular Probe, (10 mL, 12 mM solution in PBS) reagent were added and the wells mixed with a multichannel pipette. The mixtures were then incubated at 37°C in a humidified 5% CO_2_ incubator for 3h and all the media was replaced with DMSO (100 μL). The solutions were incubated at 37°C for 20 min, mixed and the absorbance was read at 570 nm by Microplate reader (Bio-RAD).

Concentration dependence of the nanoparticles. The procedure was similar to that described in the previous section. 7μg/mL venom was added into the wells and various concentrations of the nanoparticle from 1 to 150 μg/mL were added to the wells 5 min later. Culture media controls (cell and media with no venom) and the control without the addition of the nanoparticles were also run in parallel. MTT assay was used to evaluate the cell viability at 570 nm.

Calculation of the nanoparticle efficacy. Half maximal effective concentration (EC50) refers to the concentration of the nanoparticle which induces a response halfway between the baseline and the maximum after a certain exposure time. The equation is shown below.

$$Y=(Bottom)+\frac{\left(Top)-(Bottom\right)}{1+{\left(\frac{X}{EC50}\right)}^{-(Hill\;coefficient)}}$$

Where Y is the cell viability, Bottom is 0 (the lowest cell viability), Top is 100 (the highest cell viability) and the Hill coefficient gives the largest absolute value of the slope of curve. Origin 8 is used to fit a following sigmoidal function and calculate EC 50.

### In vivo protocols

Venom. The venom of *N*. *nigricollis* was purchased from Latoxan and was obtained from adult specimens of this species collected in Cameroon. Venom was stored at -20°C and was dissolved just before each experiment.

Animal experiments. Mice of the CD-1 strain of 18–20 g body weight were used throughout the study. All animals received food and water *ad libitum* and were submitted to a daily light: dark cycle of 12 h each. The protocols used in animal experiments were approved by the Institutional Committee for the Care and Use of Laboratory Animals (CICUA) of the University of Costa Rica (CICUA 82–8).

Assessment of toxicity of nanoparticles. A group of five mice received 100 μL of a nanoparticle suspension (5.5 mg/mL) by the intravenous (i.v.) route in the caudal vein. A control group of mice received 100 μL of 0.12 M NaCl, 0.04 M phosphate, pH 7.2 solution (PBS) under otherwise identical conditions. Mice were closely observed for signs of acute toxicity and changes in behavior during 4 h, and then left for 24 h to assess lethality. At this time, mice were sacrificed by CO_2_ inhalation and tissue samples from heart, liver and kidney were collected and immediately placed in fixative (3.8% formalin). After routine processing and embedding in paraffin, 4 μm sections were obtained and stained with hematoxylin-eosin for histological observation. Another group of mice received 100 μL of the nanoparticle suspension (5.5 mg/mL) intramuscularly in the right gastrocnemius muscle. Control mice received the same volume of PBS. Three hours after injection, a blood sample was collected from the tail, and the creatine kinase (CK) activity of plasma was quantified, by using a commercial kit (CK LIQUI-UV, Stanbio Lab., Texas, USA), as an index of myonecrosis [26]. At 24 h after injection mice were sacrificed by CO_2_ inhalation and a sample of the injected gastrocnemius muscle was obtained and processed for histological evaluation as described above. Another group of mice received 100 μL of a nanoparticle suspension (5.5 mg/mL) by the intradermal route of injection in the ventral abdominal region. Seventy-two hour after injection, animals were sacrificed as described, a sample of injected skin was obtained and placed in 3.8% formalin solution, and processed for histological evaluation as described above.

Inhibition of dermonecrotic activity. Mixtures containing a fixed dose of venom and variable amounts of NPs, both dissolved in PBS, were prepared as to have NP: venom ratios (w : w) of 5, 2.5, 1.25 and 0.62. Controls included venom incubated with PBS only and NPs incubated with PBS only. Mixtures were incubated at 37°C for 30 min. Then, aliquots of 100 μL of the mixtures, containing 100 μg venom, were injected intradermally, in the abdominal ventral region, into groups of five mice. This venom dose was selected because it induces a necrotic lesion in the skin of approximately 60 mm^2^ and had been previously used in the description of the skin necrosis induced by this venom [10]. Seventy-two hours after injection, mice were sacrificed by CO_2_ inhalation, their skin was removed, and the area of necrosis in the inner side of the skin was measured. Inhibition was expressed as the Median Inhibitory Ratio (IR_50_), corresponding to the NP: venom ratio at which the area of the necrotic lesion was reduced by 50% as compared to controls injected with venom alone. Skin tissue samples were then placed in fixative solution and processed for histological evaluation as described above.

In another set of experiments, groups of five mice received an intradermal injection of 100 μg venom, dissolved in 50 μL of PBS. Then, at various time intervals (0, 5, 15 and 30 min), 50 μL of NPs (5.5 mg/mL) were injected intradermally at the same site of venom injection. A control group of mice received venom and then PBS instead of NPs. After 72 h the necrotic area was assessed as described.

### Statistical analysis

The significance of the differences between mean values of experimental groups was assessed by Analysis of Variance (ANOVA), followed by Tukey test for comparing pairs of means.

### Ethics statement

The protocols used in animal experiments were approved by the Institutional Committee for the Care and Use of Laboratory Animals (CICUA) of the University of Costa Rica (CICUA 82–8). This study meets the International Guiding Principles for Biomedical Research Involving Animals of the Council of International Organizations of Medical Sciences (CIOMS)

## Results and discussion

### Abiotic approach to antivenom

Several natural and synthetic molecules have been explored as possible candidates for venom inhibitors [27–29]. Our approach differs from traditional strategies centered on immune-based neutralization or inhibition by small molecule inhibitors of variable chemical nature. We are developing abiotic synthetic polymer nanoparticle(s) (NPs) that have been engineered to sequester and neutralize several of the major toxic components of snake venoms.

The “engineering” process that is used to identify the optimal chemical composition of a NP for a target protein is called directed chemical evolution (DCE). This involves creating small libraries of NPs with different chemical compositions and screening them against the target protein. Lead candidates (those with some affinity for the protein target) are identified and taken through another round of optimization. This process has been described in detail [30].

The interactions between polymer nanoparticles (NPs) and proteins are as complex as protein-protein interactions. Binding involves combinations of weak interactions that include electrostatic, hydrogen bonding, dipole-dipole and hydrophobic interactions. The relative importance of each interaction depends upon the specific composition of NP and biomacromolecule partner. By systematically varying chemical composition of the nanoparticle we can identify functional groups that contribute to binding. A quantitative analysis of the interaction of a polysulfated carbohydrate (heparin) and NPs of different chemical composition using isothermal titration calorimetry (ITC) established that binding arises from a combination of weak interactions (hydrogen bonding, electrostatic and hydrophobic) and binding conditions (temperature, pH, buffer and ionic strength) that contribute to binding [31]. A similar analysis of the NPs and proteins in this study is scheduled for a future study.

Our first report described a synthetic polymer hydrogel NP that binds and inhibits the catalytic activity of PLA_2_, one of the important families of protein toxins found in the venom of many species of snakes [32].

When exposed to human serum alone, the NP associates with endogenous apolipoproteins, primarily apolipoprotein A-1, but upon addition of venom, the bound apolipoproteins are exchanged for venom PLA_2_ proteins, suggesting that these materials could be used as a toxin sequestrant in a complex biological setting. This NP was shown to be non-toxic in vitro, as have similar NP hydrogels in vivo [33–35]. We now report an expanded study of the use of this abiotic toxin sequestrant. We have found that a single polymer NP is effective in capturing the two most important families of protein toxins common in Elapidae snakes: PLA_2_ and 3FTX. In addition, the NP has also demonstrated in vitro and in vivo inhibition of toxic activities of *Naja nigricollis* venom, a medically relevant spitting cobra from sub-Saharan Africa which causes envenomings characterized by severe local tissue necrosis [36]. This finding offers promise that a broad-spectrum toxin sequestrant to mitigate local tissue damage may be accessible. The subcutaneous or intramuscular administration of these synthetic polymer NPs, shortly after the bite at the site of envenoming, have the potential to halt or reduce the extent of local tissue damage and prevent the systemic distribution of toxins post-envenoming.

### Elapidae snake venom screening

**Fig 2:**
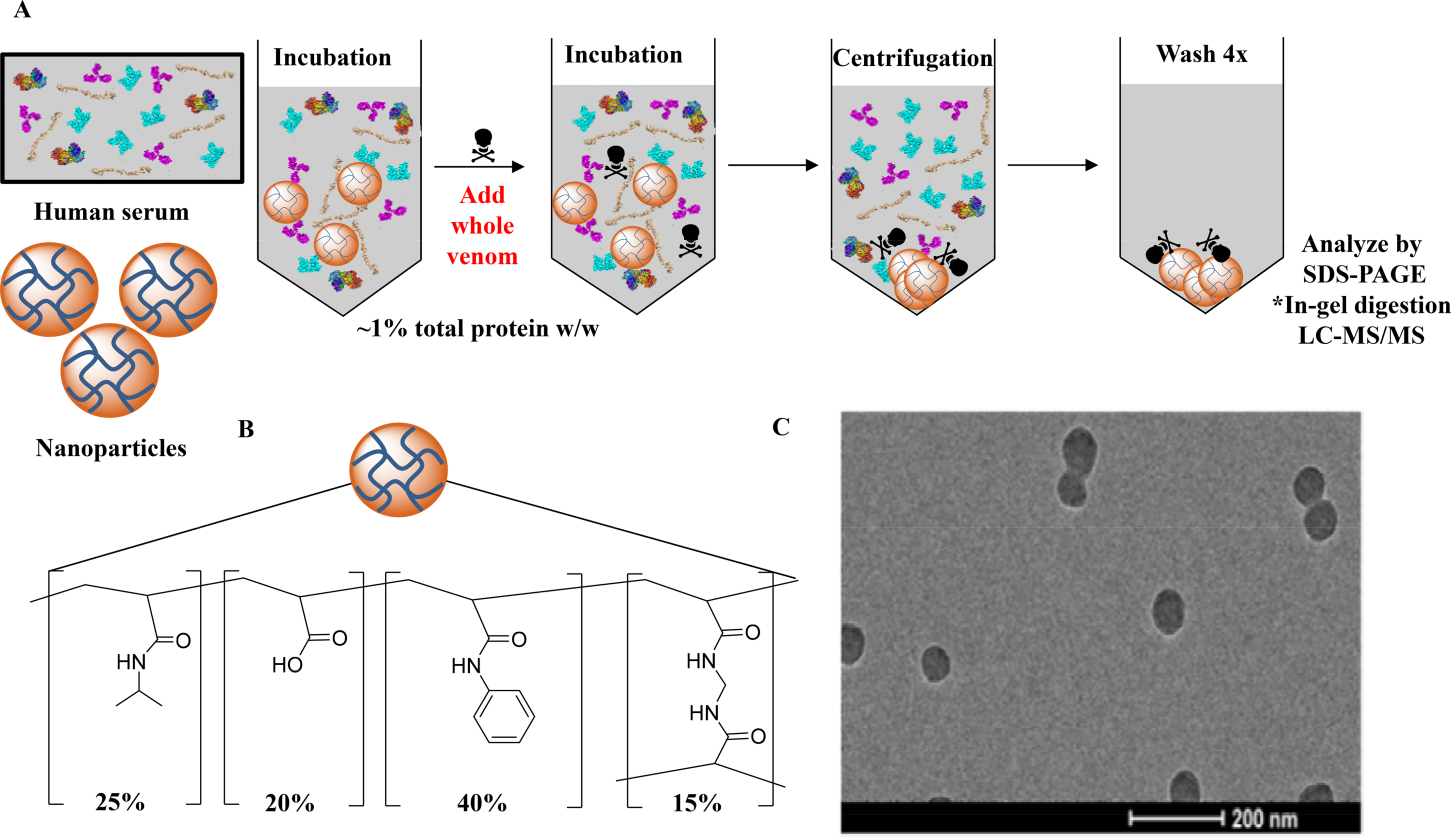
(A) Method used to analyze the binding of the synthetic NPs to various venoms. *A representative venom from a species of each genus tested was further analyzed by LC-MS/MS. (B) Composition of the synthetic NPs. (C) TEM image of the NPs (scale bar = 200 nm).

**Fig 3:**
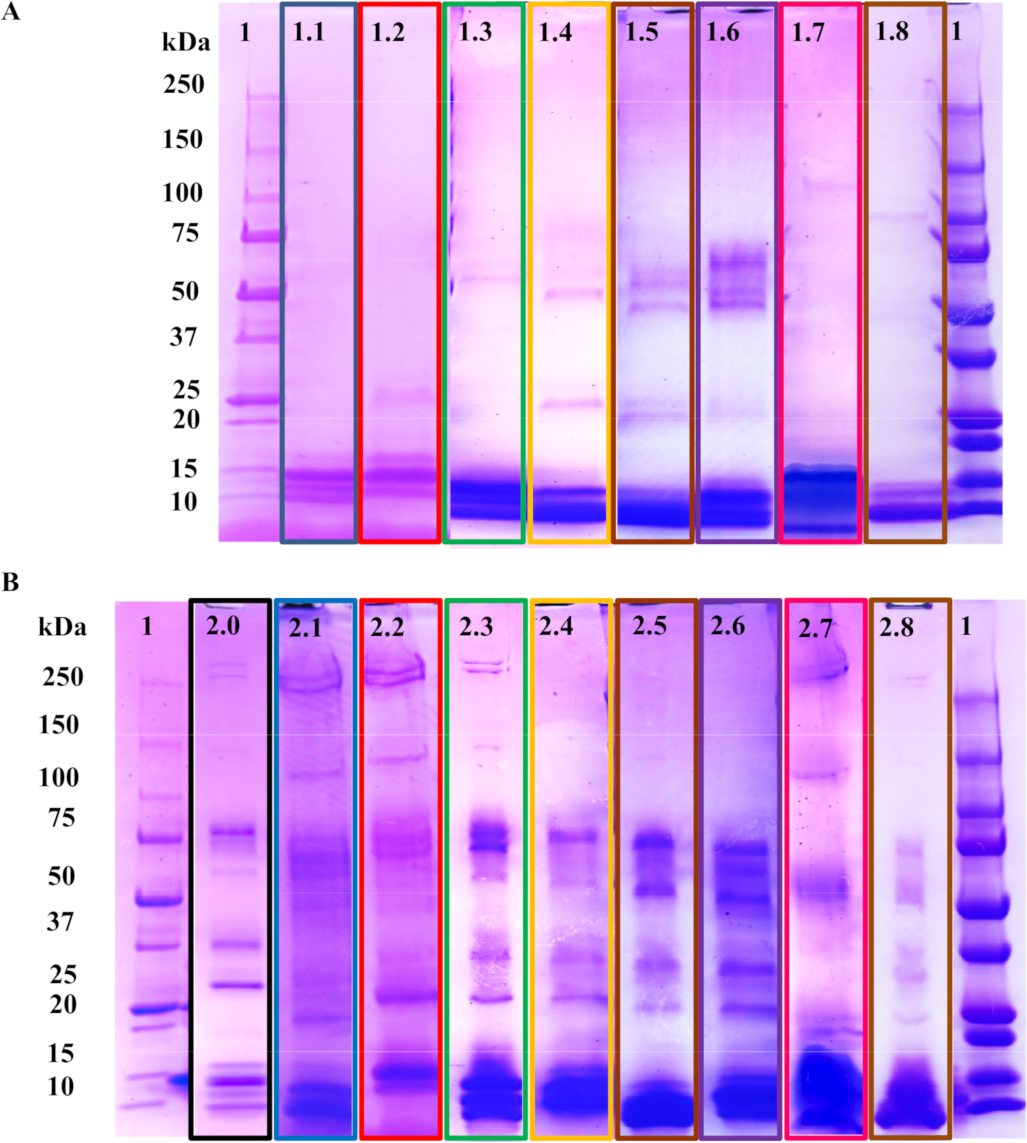
**Nanoparticle binding to elapid snake venoms in concentrated human serum** (A) Control experiments using the venom compositions of interest. (1) MW ladder. (1.1) Venom from *Naja mossambica*. (1.2) Venom from *Bungarus caeruleus*. (1.3) Venom from *Bungarus fasciatus*. (1.4) Venom from *Naja haje*. (1.5) Venom from *Naja nivea*. (1.6) Venom from *Naja melonoleuca*. (1.7) Venom from *Naja sputatrix*. (1.8) Venom from *Dendroaspis polylepis*. (B) NP selectivity results. (1) MW ladder. (2.0) Serum only control experiment. (2.1–2.8) NP selectivity results following the same venom-order described above. See S1–S10 Figs for full gel images.

Our report begins with an evaluation of the affinity of the abiotic hydrogel NP against a variety of medically relevant elapid whole snake venoms. The monodisperse NP hydrogels (176 nm, 0.044 PDI by dynamic light scattering) (Fig 2C), synthesized via precipitation polymerization with a feed ratio of 20% acrylic acid, 40% *N*-phenylacrylamide, 25% *N*-isopropylacrylamide, and 15% *N*,*N′-*methylenebis (acrylamide) (Fig 2B) was tested for toxin-selectivity in human serum by first subjecting the NP to concentrated human serum (25% (w/v), final concentration). Following a pre-incubation at 37°C, a small quantity of whole venom (250 μg/mL, final concentration) was added to the NP-serum mixture and incubated. After centrifugation and multiple washing steps, the bound proteins that constitute the *hard* protein corona [37] of the NP were examined via SDS-PAGE (Fig 2A). A representative venom extract from a species of each genus was further analyzed by LC-MS/MS to confirm that toxins found in the control experiments (Fig 3A) were also present in the NP-corona complex (Fig 3B) and to establish if the NP sequesters isoforms of both PLA_2_ and 3FTXs families.

Compared to the serum-only control experiment (Fig 3 B2.0), which shows high quantities of apolipoprotein-A1, the venom-containing experiments (Fig 3 B2.1–2.8) show that very large quantities of low-molecular weight proteins (<15 kDa) are captured by the NP. Considering that venoms themselves are dominated by proteins with MWs below 15 kDa (Fig 3 A1.1–1.8), it is likely that the proteins of that MW in the NP binding experiments are the predominant ones in the venom. To verify this claim, the samples from experiments involving *Naja mossambica* (Fig 3A1.1 and 3B2.1), *Bungarus caeruleus* (Fig 3A1.2 and 3B2.2), and *Dendroaspis polylepsis* (Fig 3A1.8 and 3B2.8) venoms were analyzed by tandem mass spectrometry via an in-gel digestion with trypsin. The detected peptide fragments were analyzed using the uniprot database of known venom proteins for each species [38]. Although many of these venoms and those of closely related species (eg., *Dendroaspis* taxa [39]; *N*. *melanoleuca* [40]; *B*. *caeruleus* [41]; *N*. *sputatrix* [42]; *N*. *haje* [43]; *B*. *fasciatus* [44]) have been characterized and some of their toxins sequenced previously, the intraspecies variability of venoms does not guarantee that all toxin isoforms found in the database are present in appreciable amounts in our venom samples, and vice versa. Furthermore, many protein isoforms share high sequence identity which can render protein identification via trypsin digestion difficult. Nevertheless, proteomic analysis will at the very least establish whether the proteins in the venom control (Fig 3A) are found in the NP protein corona (Fig 3B).

*Naja mossambica* venom, which is largely composed of cytotoxins from the 3FTX family and PLA_2_s, can induce extensive cutaneous necrosis in its victims [36]. While the number of 3FTXs and PLA_2_ isoforms expressed in the venoms of this species likely varies ontogenetically and geographically, seven 3FTXs and three PLA_2_ isoforms have been attributed to this snake venom via the Swiss-Prot database. Nine of the toxins were detected in the venom only control experiment (Fig 3 A1.1) and eight of those toxins were found in the NP and serum containing experiment (S1 Table). The lack of neurotoxin 2 (3S13_NAJMO) in the NP hard-corona complex may be attributed to the overall low concentration of the neurotoxin in the venom. Regardless, this experiment verified that the NP sequestered nearly all the isoforms of the 3FTXs and PLA_2_ toxins in *N*.*mossambica* venom in the presence of serum proteins.

A similar proteomic analysis was performed on the venom–serum uptake experiments from *Bungarus caeruleus*, the common krait from India. Envenoming by *B*. *caeruleus* results in severe neurotoxicity [45]. A major component of this venom is the PLA_2_ heterodimeric neurotoxin β-bungarotoxin, which features a PLA_2_ covalently attached to a Kunitz-type serine protease inhibitor [46]. LC-MS/MS analysis of the venom control (Fig 3 A 1.2) revealed six proteins: Four monomeric PLA_2_s and two PLA_2_ toxins that are a component of β-bungarotoxin. LC-MS/MS analysis of the NP-*B*. *caeruleus* experiment (Fig 3B 2.2) revealed that all six proteins were present in the NP protein corona (S2 Table).

The third venom, *Dendroaspis polylepsis*, which is known for its fast-acting neurotoxic effect [36], is well characterized and therefore a good candidate for proteomic assessment. Analysis of the venom control (Fig 3A1.8) revealed eight unique 3FTXs and four different Kunitz-type serine protease inhibitors which in this venom correspond to dendrotoxins, a group of neurotoxins that block the voltage-sensitive potassium channels in neurons [47]. Seven of the eight 3FTXs were found in the NP protein corona (Fig 3B2.8), Moreover, three of the four dendrotoxins were also found in the NP corona along with an acetylcholinesterase (S3 Table). This underscores that NPs are able to bind not only 3FTXs and PLA_2_s, but also dendrotoxins, which belong to a different structural family of neurotoxic venom proteins. This has evident therapeutic implications when dealing with mamba snake venoms.

Despite differences in toxin composition across different species, these venom-selectivity experiments support the hypothesis that the nanoparticles are able to bind to different structural determinants in different groups of toxins from elapid snake venoms over abundant serum proteins. While the results indicate broad-sequestration of certain important venom toxins of elapid sakes by the NP, this does not necessarily translate to broad-neutralization of these toxins. To this point, we focused on the neutralization of necrotizing toxins, whose neutralization by antivenoms is limited [10]. We identified venom from the African spitting cobra, *Naja nigricollis*, as an ideal candidate for this study since envenoming from this species is responsible for many cases in sub-Saharan Africa resultant in sequelae secondary to local cutaneous necrosis [36, 48].

### Selectivity for *Naja nigricollis* venom

The pathophysiology observed in envenomings caused by *N*.*nigricollis* results from a synergistic effect involving numerous PLA_2_ and 3FTX isoforms [10]. 3FTXs, which comprise 73% of the venom of *N*. *nigricollis* [49], are the major toxin family responsible for its toxicity [10]. We first evaluated the protein corona of the NP in the presence of human serum and *N*. *nigricollis* venom as described in the previous section.

**Fig 4:**
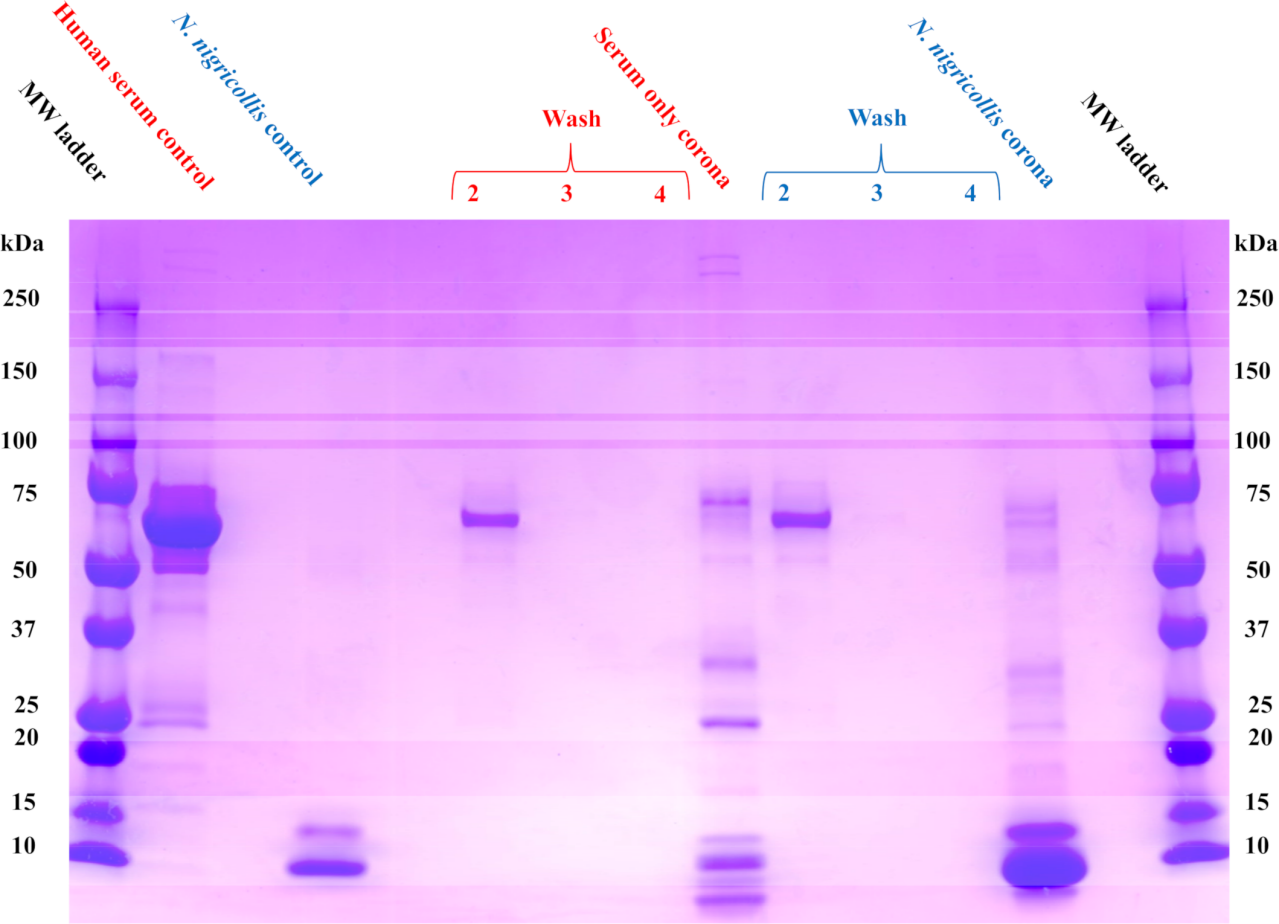
NP selectivity in human serum (red) and in human serum + *N*. *nigricollis* venom (blue). In the presence of serum alone, the NP favorably associates with proteins of varying molecular weights (Serum only corona band). A distinct change in the distribution and a new dominant molecular weight band (<15 kDa) in the *N*. *nigricollis* venom-containing corona band. Proteins that comprised this NP corona were identified via in gel digestion followed by proteomic identification.

Compared to the serum only control experiment, the addition of venom after an initial serum incubation resulted in a dominant protein band in the molecular weight range observed in the venom control (<15 kDa) (Fig 4). In the presence of serum alone, the NP favorably associates with proteins of varying molecular weights. However, there is a distinct change in the distribution and a new dominant molecular weight band (<15 kDa) in the venom-containing corona experiment. Proteins that comprised the NP corona were identified via in gel digestion followed by proteomic identification. Unlike the previous proteomic studies, venom from *N*. *nigricollis* only has four toxins attributed to it in the UniProt database: one full-length PLA_2_, two 30 amino acid fragments of PLA_2_ and a 51 amino acid peptidase inhibitor called nawaprin. We therefore extended the database to include toxin sequences from the closely related species *Naja pallida* which includes two 3FTXs (cytotoxin and neurotoxin). Analysis confirmed that all the toxins observed in the *N*. *nigricollis* venom control were also observed in the NP protein corona (S4 Table). These results were quite similar to the proteomics analysis of the related *N*. *mossambica* venom-selectivity study in the previous section and reinforce the concept that the NP is able to bind the most relevant toxin families in these snake venoms.

### In vitro inhibition of *Naja nigricollis* venom

The toxin uptake studies with whole venom from the spitting cobra *N*. *nigricollis* and other elapid snakes in the presence of serum proteins were encouraging. However, sequestration does not necessarily translate to inhibition of toxicity. *N*. *nigricollis* induces cytotoxicity and produces substantial myonecrosis. To establish if the NP is capable of neutralizing myonecrosis in vitro, we designed a cell viability study of rat skeletal muscle cells (L6) in the presence of *N*. *nigricollis* venom and the NP.

**Fig 5:**
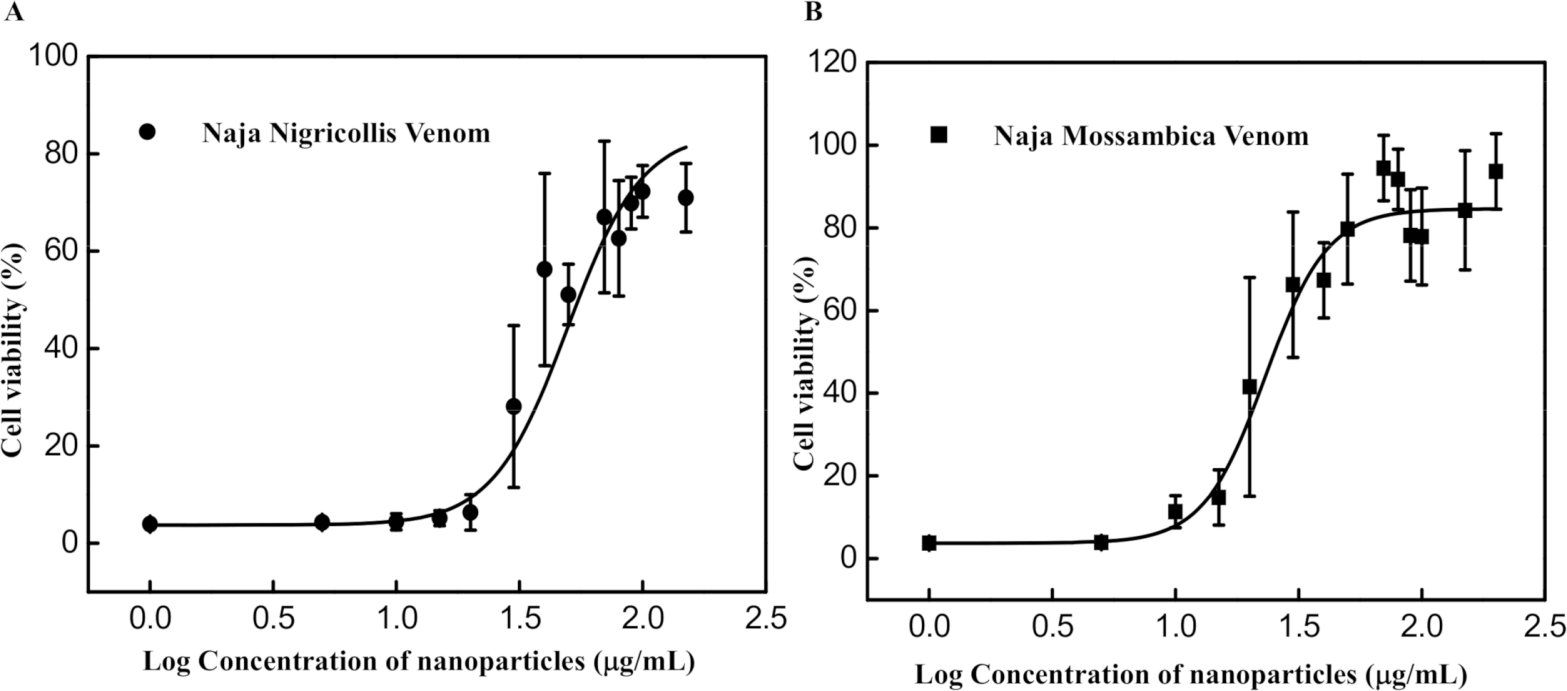
**Dose-response curve of inhibition of cytotoxicity in L6 muscle cells by the NP against administration of 7 μg/mL** of (A) *N*. *nigricollis* venom and (B) 7 **μ**g/mL of *N*. *mossambica* measured 14 h after exposure to the cells (n = 4–5).

The MTT assay was used to visualize metabolically active cells in the evaluation of the inhibition of venom induced cytotoxicity by the NP. S11 Fig shows the cell viability of L6 cells incubated with *N*. *nigricollis* venom (1–30 μg/mL, n = 4–5) in the presence and absence of 80 μg/mL NP. Results indicate that the NP is effective at neutralizing the toxicity from *Naja nigricollis* venom. A concentration of 7 μg/mL of *N*. *nigricollis* venom resulted in almost 100% cytotoxicity of L6 cells in the absence of the NP. This concentration was selected for further experiments to study the dose-response curve of the NP (Fig 5). The results show a dose-dependent relationship between NP concentration and cell survival (Fig 5). The first concentration to have a significant effect on cell survival is 49.6 μg/mL which resulted in 50% cell survival (half maximal effective concentration, EC_50_). In addition, a ratio of venom: NP (w/w) ranging from 1:5 to 1:7 resulted in near complete inhibition of cytotoxicity. This was substantially more potent than the reported ratio of South African antivenom (SAIMR) which required a ratio greater than 1:150 venom: antivenom (w/w) to completely inhibit *N*. *nigricollis* venom-induced cytotoxicity in L6 cells [50].

We also evaluated the efficacy of the NP to inhibit cytotoxicity of *Naja mossambica* venom, another member of the Elapidae snake family to ensure that this result was not exceptional (Fig 5B). The calculated EC_50_ is 23.2 **μ**g/mL and a ratio ranging from 1:6 to 1:7 venom: NP (w/w) brought about complete neutralization of *N*. *mossambica* venom toxicity in L6 cells. The NPs have a much higher capacity than the reported ratio of SAIMR which required a ratio greater than 1:500 venom: antivenom (w/w) to completely suppress cytotoxicity induced by this venom [50].

### In vivo studies with the venom of *Naja nigricollis*

Both selective uptake studies with whole venom and the in vitro results suggest the NP functions as a sequestrant for the major protein toxins of elapid snakes. We proceeded with an evaluation of the ability to mitigate in vivo the necrotic effects of venom of the African spitting cobra *N*. *nigricollis*. In contrast with envenomings by the majority of cobras (*Naja* sp), which are characterized by neurotoxic manifestations, those inflicted by *N*. *nigricollis* are characterized by a prominent cytotoxic effect, clinically manifested by blistering and local cutaneous necrosis, which often end up in permanent sequelae such as chronic ulceration, hypertrophic scars, keloid formation, blindness and, in some cases, malignant transformation [11, 36]. Previous in vitro studies and our current observations demonstrated the ability of NPs to sequester and inhibit PLA_2_s and cytotoxins of the 3FTX family from the venom of the spitting cobra *Naja mossambica* [32], and a closely related species, *N*. *nigricollis*. We next evaluated the ability of the NP to inhibit the necrotic activity of the venom of *N*. *nigricollis* in skin. To this end, a mouse experimental model developed in one of our laboratories [10] was used to assess the efficacy of NPs.

### Assessment of toxicity of NPs

**Fig 6:**
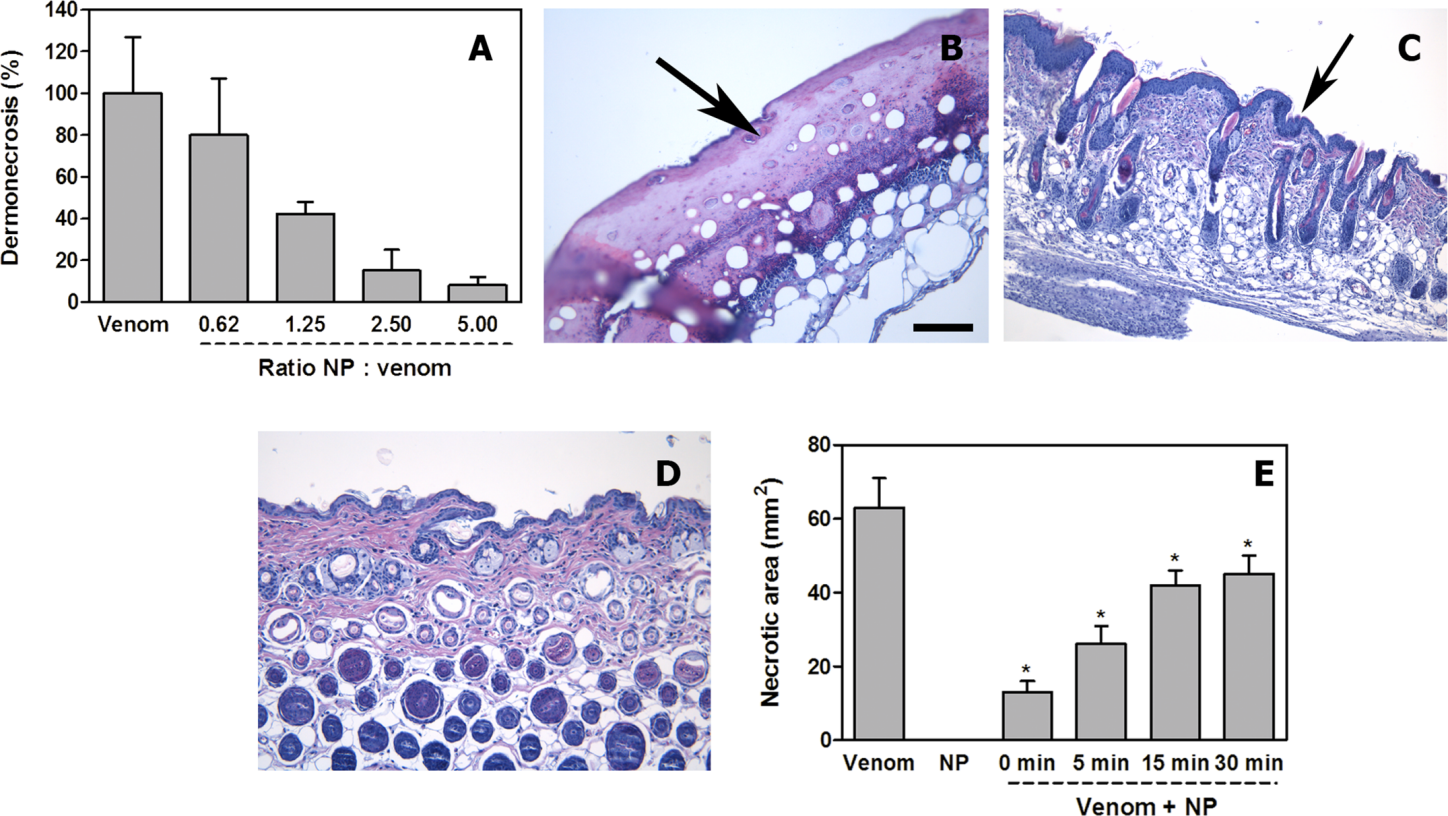
Inhibition of dermonecrotic activity of *N*. *nigricollis* venom by NPs. (A) A fixed amount of venom was incubated with variable amounts of NPs to attain several ratios. Controls included venom incubated with saline solution instead of NPs. Upon incubation, aliquots of the mixtures, containing 100 μg venom, were injected intradermally in mice. Seventy-two hours after injection, the areas of necrotic lesions in the inner side of the skin were measured. Necrosis was expressed as percentage, 100% corresponding to the necrotic area (60 mm^2^) in mice receiving venom alone. NPs inhibited, dose-dependently, the dermonecrotizing activity of the venom. (B, C, D) Light micrographs of sections of the skin of mice 72 h after injection of 100 μg *N*. *nigricollis* venom incubated with saline solution (B), with NPs at a NP: venom (w : w) ratio of 5.0 (C), or after injection of NPs alone (D). In (B) there is ulceration, with loss of epidermis and the formation of a hyaline proteinaceous material (arrow); skin appendages are absent and there is a prominent inflammatory infiltrate. In (C) NPs inhibited the ulcerative effect, evidenced by the presence of epidermis (arrow) and skin appendages, whereas an inflammatory infiltrate is observed in the dermis. Skin injected with NPs alone (D) shows a normal histological pattern. Hematoxylin-eosin staining. Bar represents 100 μm. (E) Inhibition of dermonecrosis by *N*. *nigricollis* venom in experiments involving intradermal injection of 100 μg venom followed by the injection of NP suspension (5.5 mg/mL), in the same region of venom injection, at various time intervals. A significant reduction in the extent of dermonecrosis was observed (* p < 0.05), although the extent of inhibition decreased with time. NP alone did not induce skin damage.

Before assessing the ability of NPs to inhibit venom in vivo, the potential toxicity of NPs was studied. Mice receiving 100 μL of NPs (5.5 mg/mL) by the intravenous route did not show any evidence of toxicity nor any change in their behavior, as compared to mice receiving PBS. All of them survived the 24 h observation period. Mice injected intramuscularly in the gastrocnemius with 50 μL of NPs did not show any problem for mobilization, and the plasma CK activity was not increased as compared to the plasma CK activity of mice injected with PBS (302 ± 352 U/L and 206 ± 120 U/L, respectively; p > 0.05) thus indicating lack of myotoxicity. Moreover, intradermal injection of NPs did not cause any macroscopic effect in the skin. Histological assessment of tissue sections from liver, kidneys and heart (after intravenous injection), gastrocnemius muscle (after intramuscular injection), and skin (after intradermal injection) showed a normal microscopic appearance (Fig 6D; S12 Fig) which did not differ from that of tissue sections obtained from mice injected with PBS. Thus, under our experimental conditions, NPs did not induce toxicity in mice.

### Inhibition of dermonecrosis

The NPs inhibited dose-dependently the dermonecrotic activity of *N*. *nigricollis* venom, as visually assessed by the reduction or abrogation of the necrotic area (Fig 6A; Supplementary S13 Fig). The estimated IR_50_ was 1.1 (NP: venom, w:w). Histological assessment confirmed the inhibition of skin necrosis. Tissue from mice injected with venom incubated with PBS showed a characteristic necrotic pattern, similar to that described by Rivel et al [10]. These lesions are characterized by a central dark necrotic core and a peripheral whitish ring. Necrosis is characterized by loss of epidermis and of skin appendages, together with the accumulation of a hyaline fibrinoid material, together with an abundant inflammatory infiltrate with predominance of polymorphonuclear leucocytes (Fig 6B). In contrast, skin samples from mice receiving venom and NPs showed preservation of the epidermis and of the dermal structure and skin appendages, i.e. inhibition of necrosis. However, the epidermis had an increased number of epithelial cell layers and there was an inflammatory infiltrate in the dermis (Fig 6C), both of which reveal an ongoing inflammatory process in the absence of necrosis. Tissue from mice injected with NPs alone showed a histomorphology indistinguishable from that of mice receiving PBS alone (Fig 6D).

When inhibition experiments were performed by first injecting the venom and then, at various time intervals, the NPs, dermonecrosis was significantly reduced in all cases, although inhibition diminished as time lapse between envenoming and treatment increased (Fig 6E).

These results establish that an abiotic synthetic polymer NP, engineered to bind to and modulate the activity of a diverse array of PLA_2_ and 3FTX isoforms found in Elapidae snake venoms, inhibit dose-dependently the dermonecrotic activity of *N*. *nigricollis* venom, the most important clinical manifestation of envenomings by African spitting cobras. These two families of protein toxins share features that are associated with their ability to bind and disrupt cell membrane targets, thus allowing for NP sequestration that arises from a mechanism that differs from immunological (epitope) selection.

## Supporting information

S1 Fig NP selectivity experiment for *Bungarus fasciatus* venom (blue) and *Naja haje* venom (red) using 1% (w/w) venom in 25% human serum. (TIF)

S2 Fig NP selectivity experiment for *Naja nivea* venom (blue) and *Naja melanoleuca* venom (red) using 1% (w/w) venom in 25% human serum. (TIF)

S3 Fig NP selectivity experiment for *Naja sputatrix* venom (red) using 1% (w/w) venom in 25% human serum. (TIF)

S4 Fig NP selectivity experiment for *Dendroaspis polylepis* venom (blue) using 1% (w/w) venom in 25% human serum. (TIF)

S5 Fig NP selectivity experiment for *Naja mossambica* venom (red) using 1% (w/w) venom in 25% human serum. (TIF)

S6 Fig NP selectivity experiment for *Bungarus caeruleus* venom (red) using 1% (w/w) venom in 25% human serum. (TIF)

S7 Fig Gel bands selected for trypsin digestion and LC-MS/MS proteomics analysis for *Naja mossambica* selectivity experiment. (TIF)

S8 Fig Gel bands selected for trypsin digestion and LC-MS/MS proteomics analysis for *Bungarus caeruleus* selectivity experiment. (TIF)

S9 Fig Gel bands selected for trypsin digestion and LC-MS/MS proteomics analysis for *Dendroaspis polylepis* selectivity experiment. (TIF)

S10 Fig Gel bands selected for trypsin digestion and LC-MS/MS proteomic analysis for *Naja nigricollis* selectivity experiment. (TIF)

S11 Fig The cell viability of L6 cells incubated with *Naja nigricollis* venom (1–30 μg/mL, n = 4–5) in the presence (black square) and absence (black circle) of 80 μg/mL nanoparticle, respectively. (TIF)

S12 Fig **Light micrographs of sections from heart** (A), liver (B), kidney (C) and skeletal muscle (D) from mice injected with nanoparticles. Mice (18–20 g body weight) were injected with either 100 μL of nanoparticles in saline solution (5.5 mg/mL) by the intravenous (i.v.) route in the caudal vein, or intramuscularly (i.m.) in the right gastrocnemius. Mice injected i.v. were sacrificed at 24 h, and samples of heart, liver and kidneys were obtained. Mice injected i.m. were sacrificed at 24 h, and a sample of the injected gastrocnemius muscle was obtained. Tissues were fixed in 3.7% formalin solution and processed routinely for embedding in paraffin. No histopathological alterations are observed in any of the tissues. Hematoxylin-eosin staining. Bar represents 100 μm.
(TIF)

S13 Fig Macroscopic assessment of the inhibition of dermonecrotic activity of *N*. *nigricollis* venom by NPs. Venom and NPs were mixed at a NP: venom ratio of 5.0 (w/w) and incubated for 30 min at room temperature. Controls included venom incubated with saline solution (venom) and NPs incubated with saline solution (nanoparticles). Then, aliquots of each mixture were injected intradermally in the ventral abdominal region of mice (18–20 g body weight). After 72 h, animals were sacrificed, their skin removed and the inner side of the skin observed for the presence of necrosis. A dark area of necrosis is observed only in the skin of mice receiving venom incubated with saline solution. Dermonecrosis was totally abrogated by the NPs, whereas NPs incubated with saline did not induce any effect.
(TIF)

S1 Table Identification of toxins in *Naja mossambica* venom experiment using digested SDS-PAGE gel bands depicted in S7 Fig. (TIF)

S2 Table Identification of toxins found in *Bungarus caeruleus* experiment using digested SDS-PAGE gel bands depicted in S8 Fig. (TIF)

S3 Table Identification of toxins found in *D*. *polylepsis* experiment using digested SDS-PAGE gel bands depicted in S9 Fig. (TIF)

S4 Table Identification of toxins found in *Naja nigricollis* experiment using digested SDS-PAGE gel bands depicted in S10 Fig. (TIF)

S1 File The excel files for each set of MS-MS experiments. Note the tabs on the bottom of the files, which correspond to the gel band (peptide summary and protein summary—2 tabs per gel band). In every instance the uniprot database corresponding to the snake species of interest was included in the search. However, the total database used in the search varied. Sometimes the human proteome was included and other times a variety of snake venom sequences were included. In the protein summary section the proteins corresponding to the actual species of interest are highlighted.
(XLSX)
