# Supplementary figures and images for: Engineered nanoparticles bind elapid snake venom toxins and inhibit venom-induced dermonecrosis
Source: PLoS Negl Trop Dis. 2018 Oct 4;12(10):e0006736. doi: 10.1371/journal.pntd.0006736 (PMC6171825; doi:10.1371/journal.pntd.0006736)

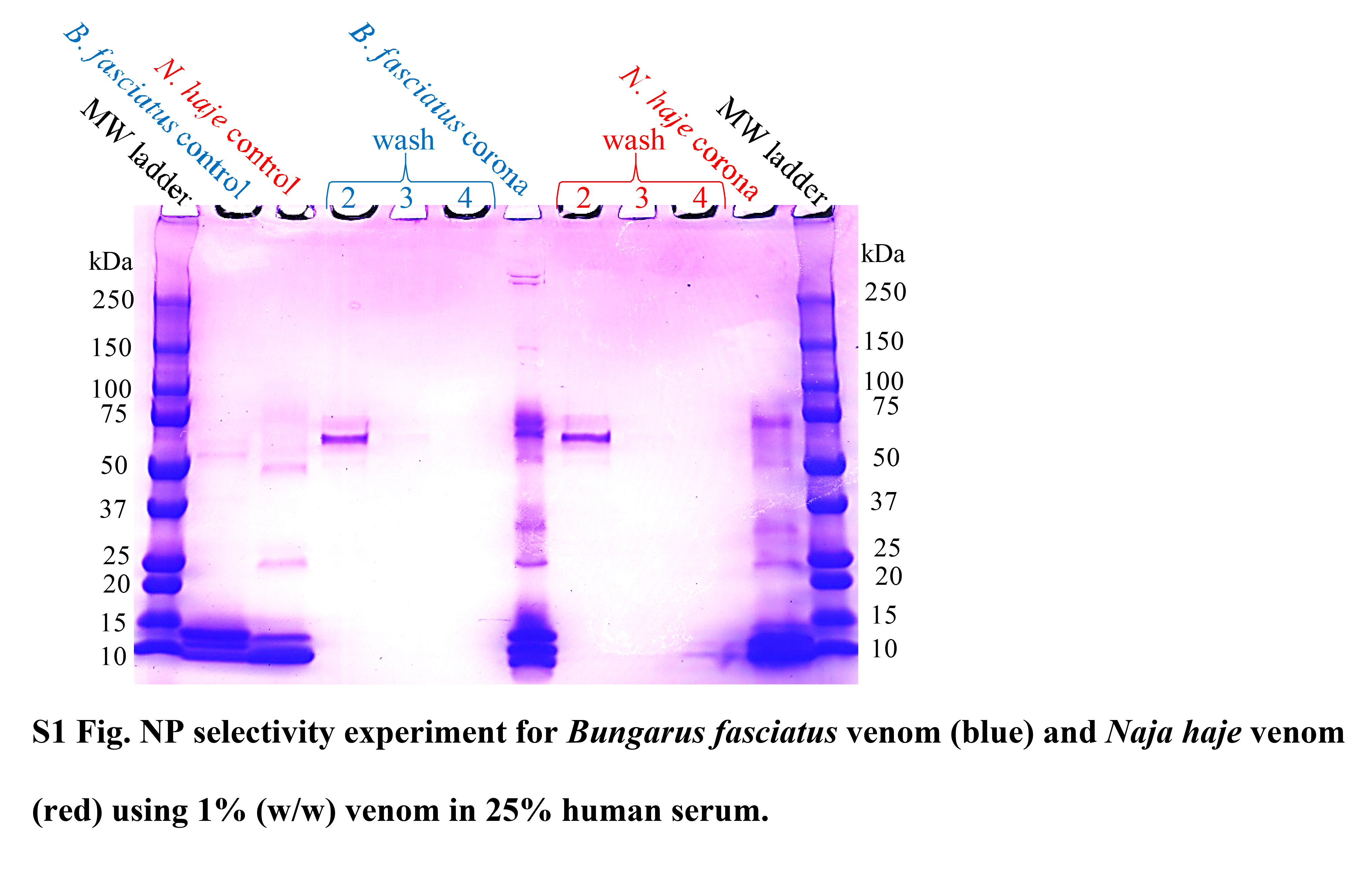

Supplement: S1 Fig — (TIF) [file pntd.0006736.s001.tif]

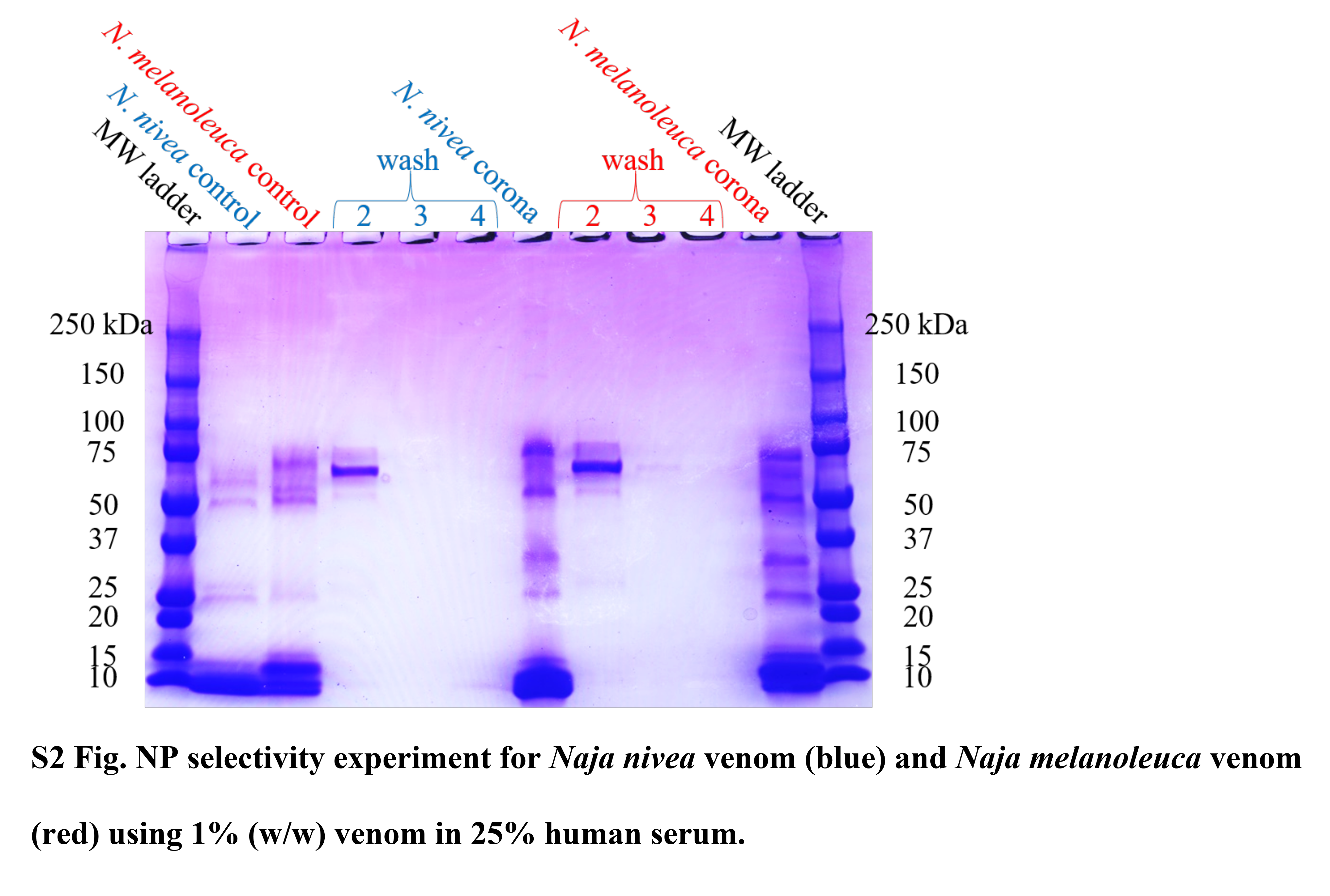

Supplement: S2 Fig — (TIF) [file pntd.0006736.s002.tif]

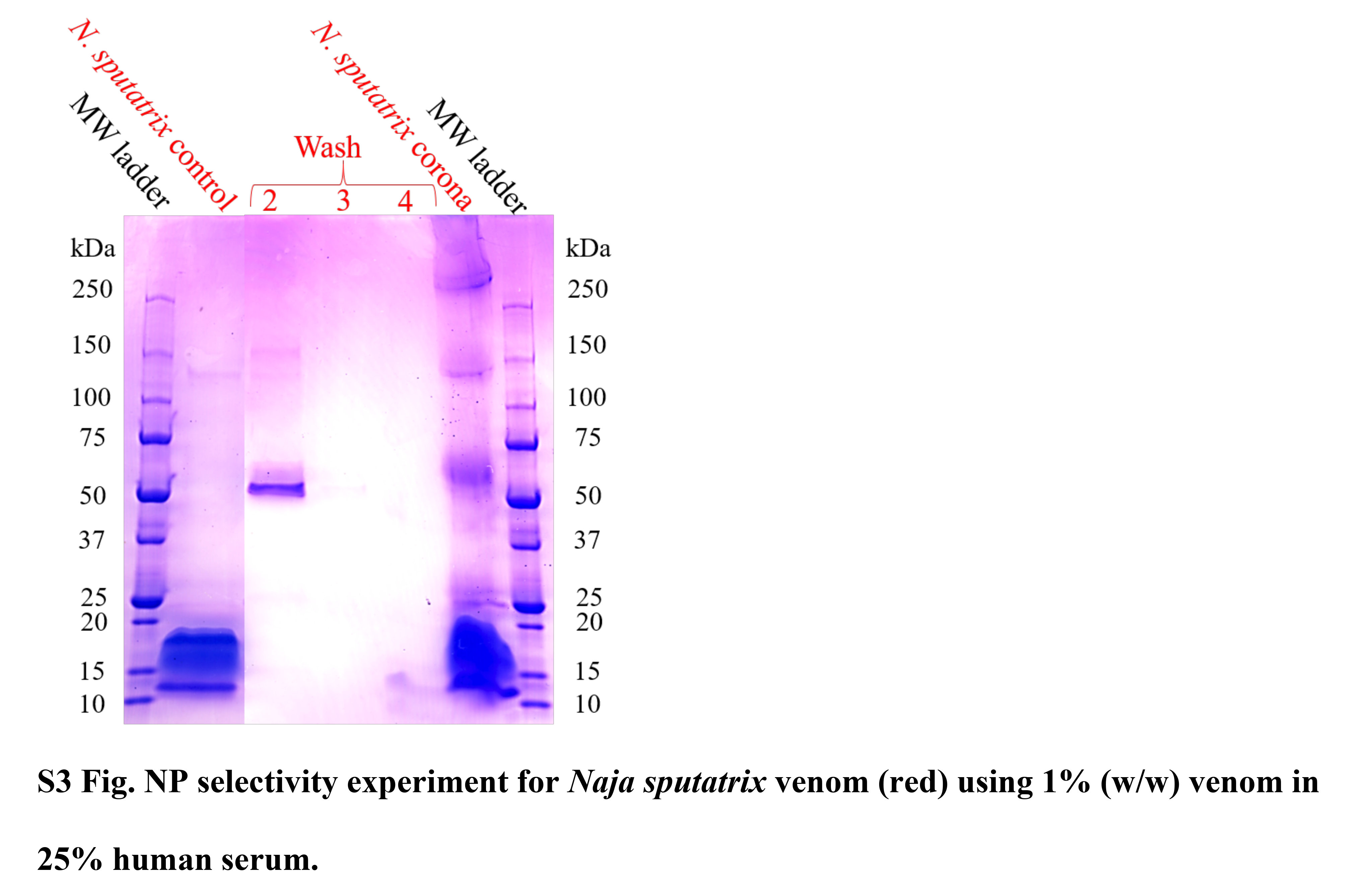

Supplement: S3 Fig — (TIF) [file pntd.0006736.s003.tif]

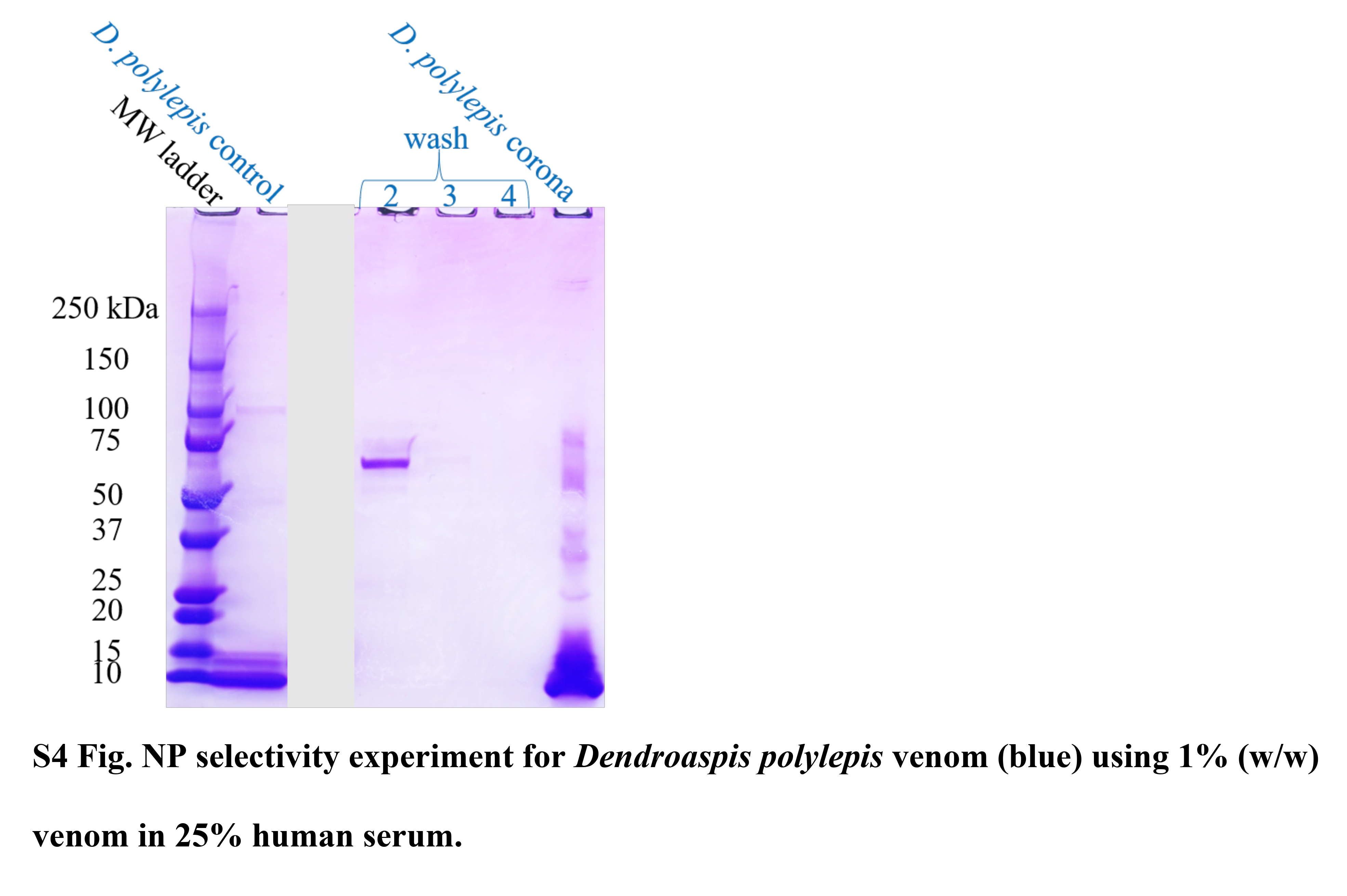

Supplement: S4 Fig — (TIF) [file pntd.0006736.s004.tif]

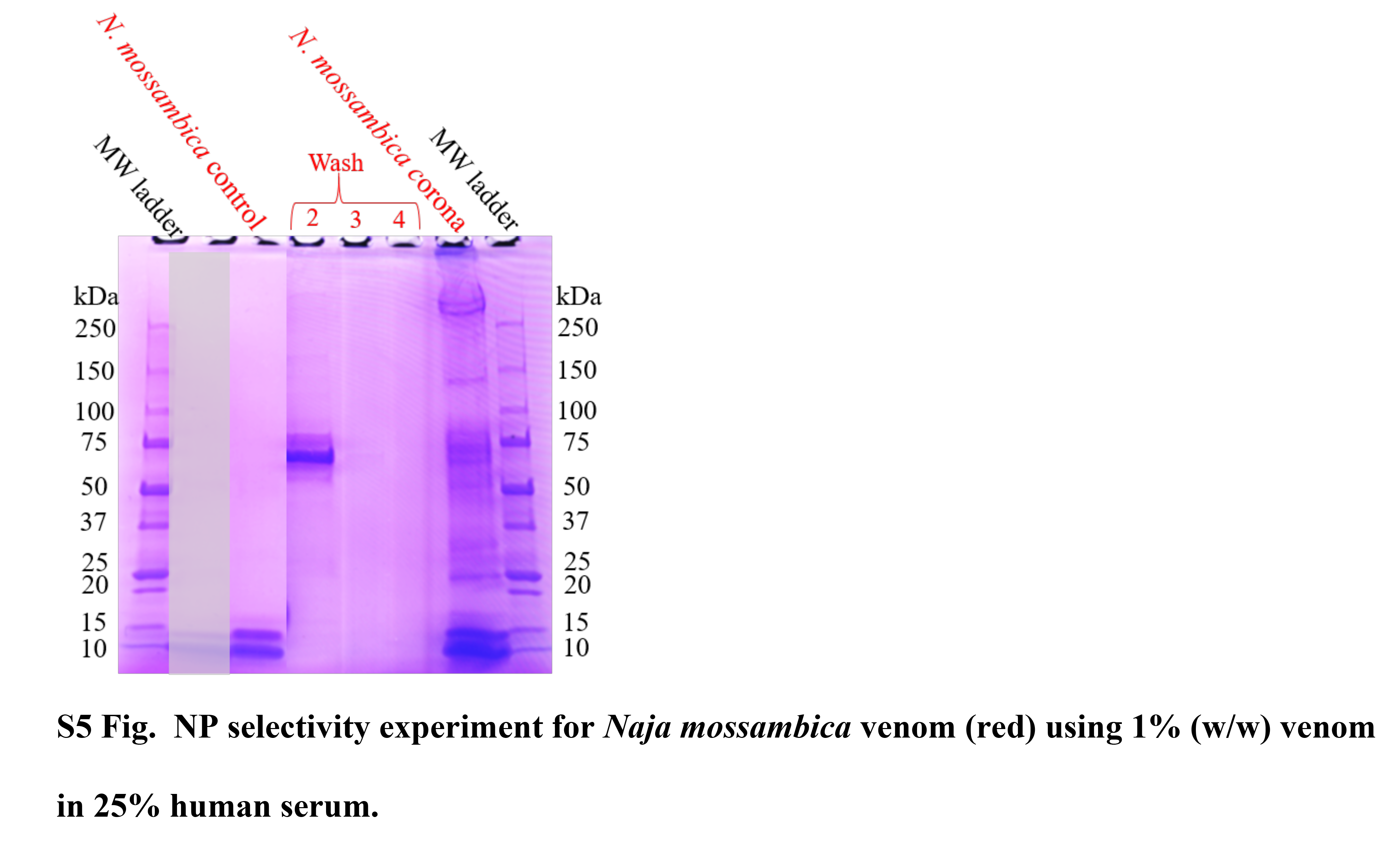

Supplement: S5 Fig — (TIF) [file pntd.0006736.s005.tif]

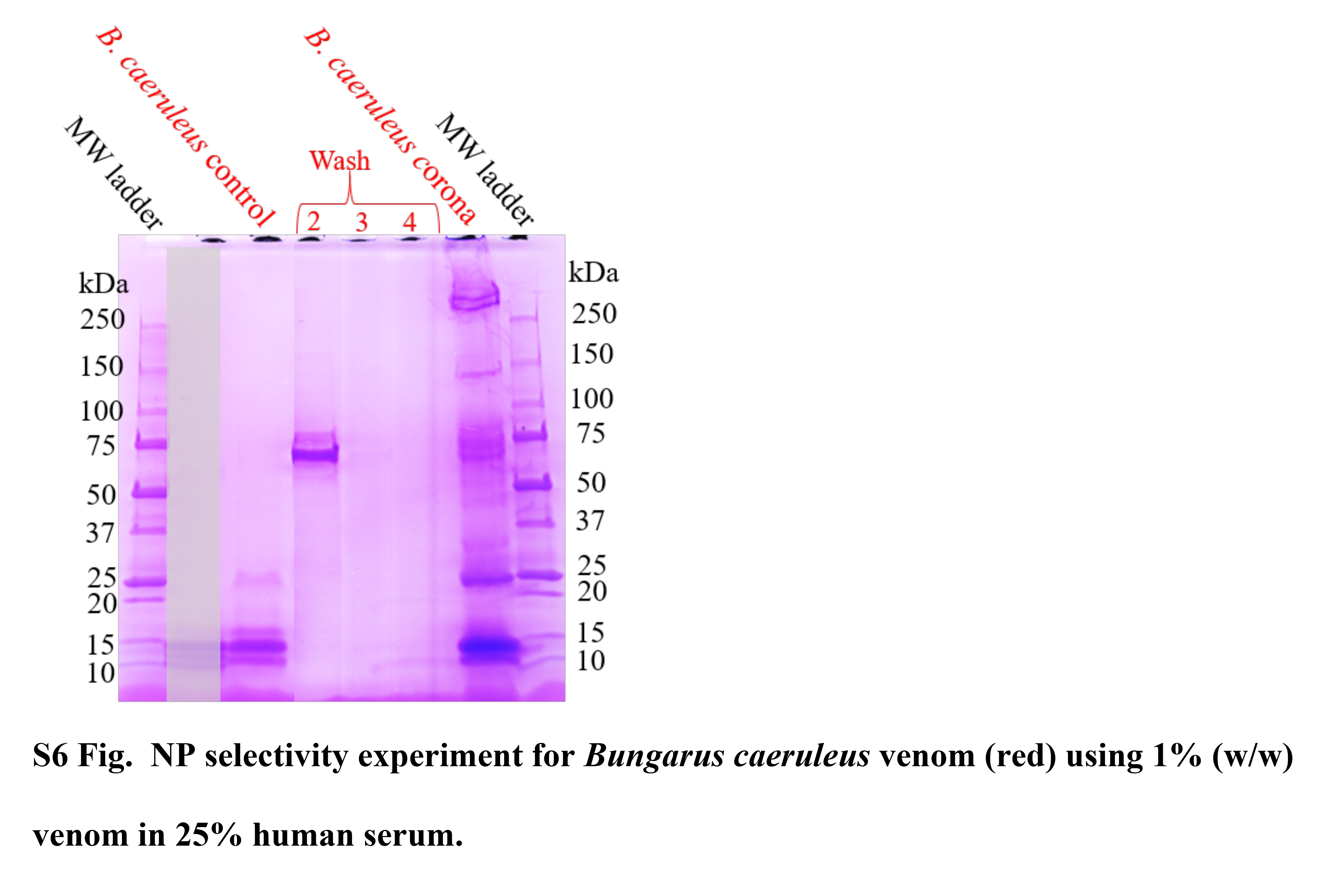

Supplement: S6 Fig — (TIF) [file pntd.0006736.s006.tif]

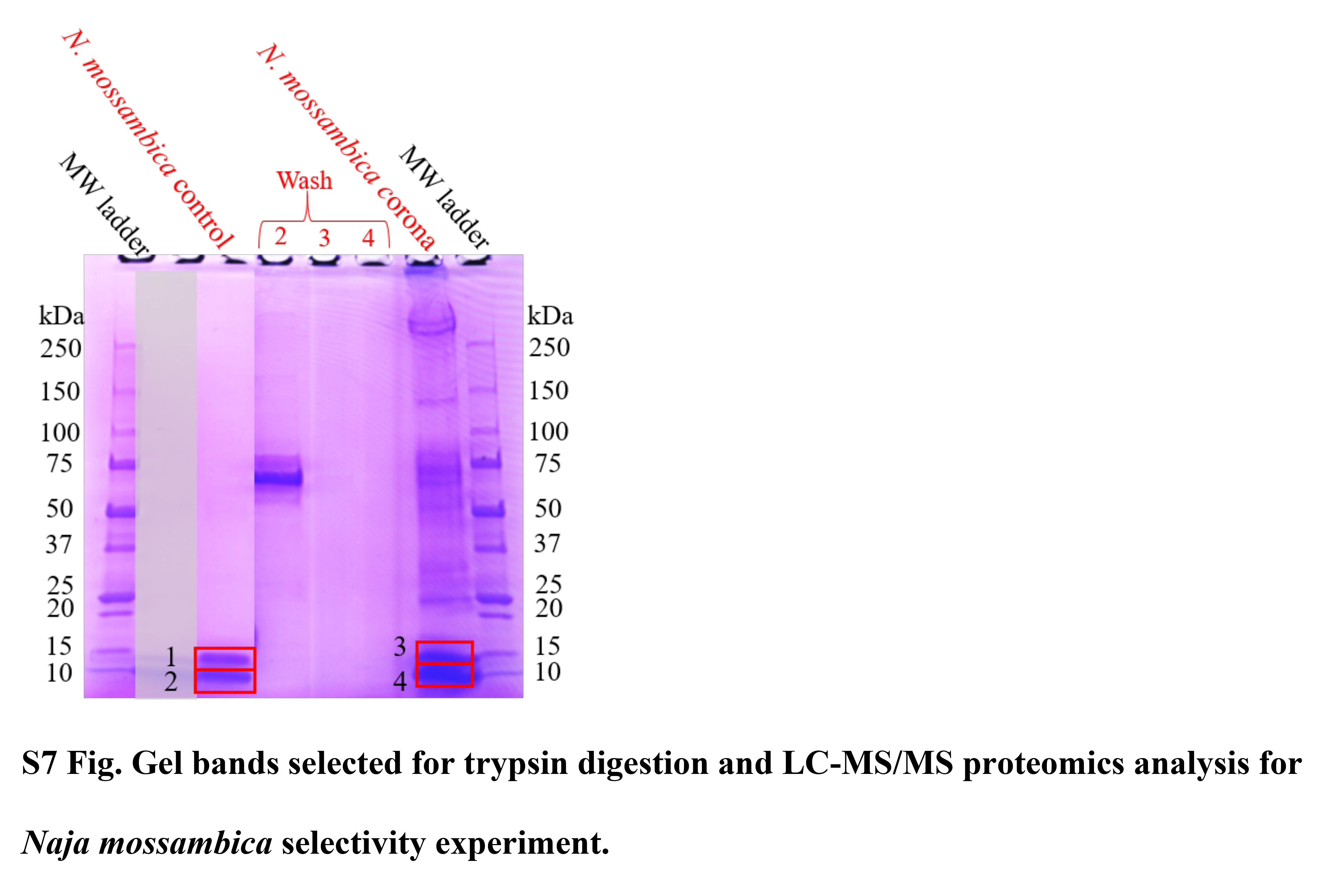

Supplement: S7 Fig — (TIF) [file pntd.0006736.s007.tif]

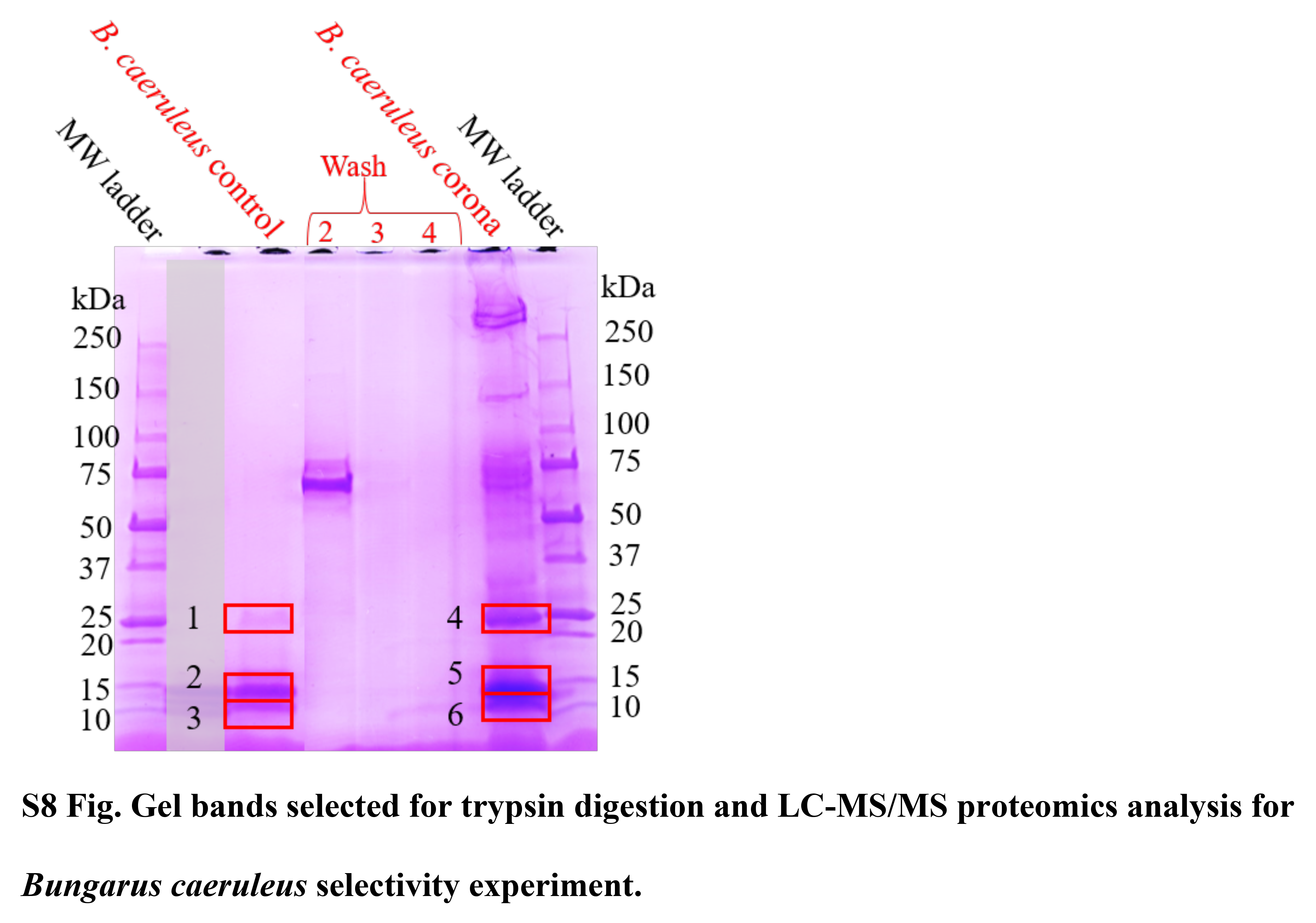

Supplement: S8 Fig — (TIF) [file pntd.0006736.s008.tif]

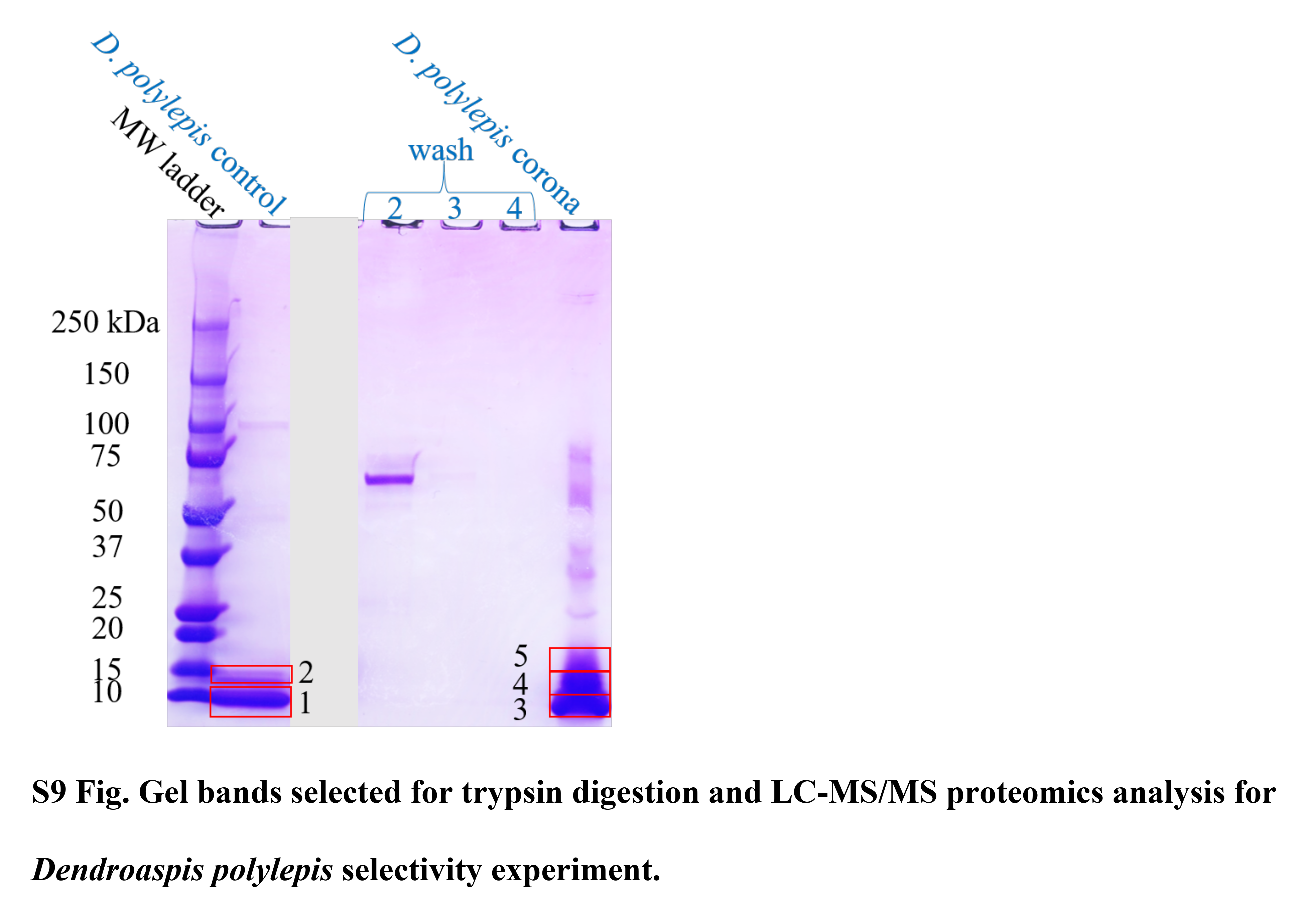

Supplement: S9 Fig — (TIF) [file pntd.0006736.s009.tif]

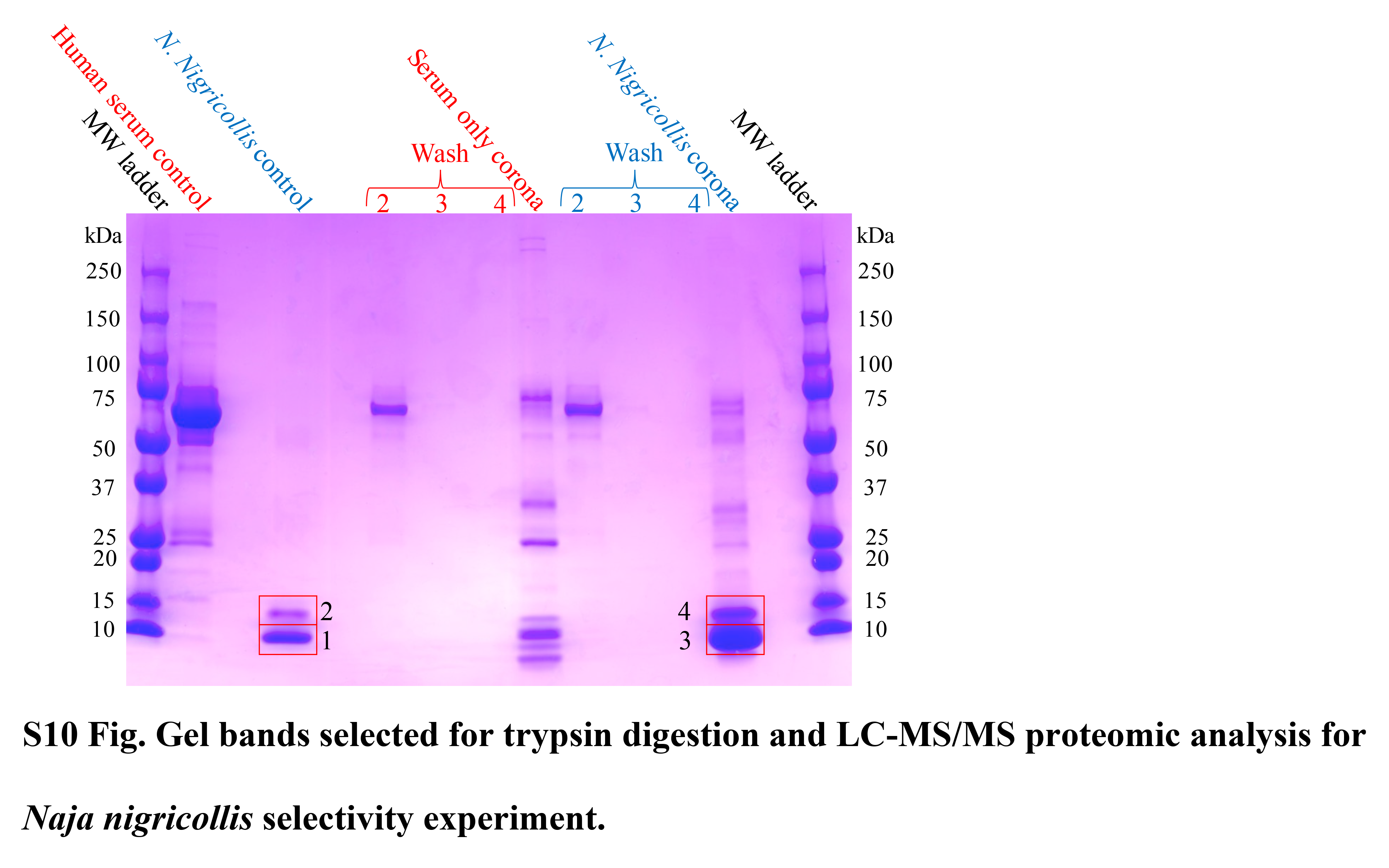

Supplement: S10 Fig — (TIF) [file pntd.0006736.s010.tif]

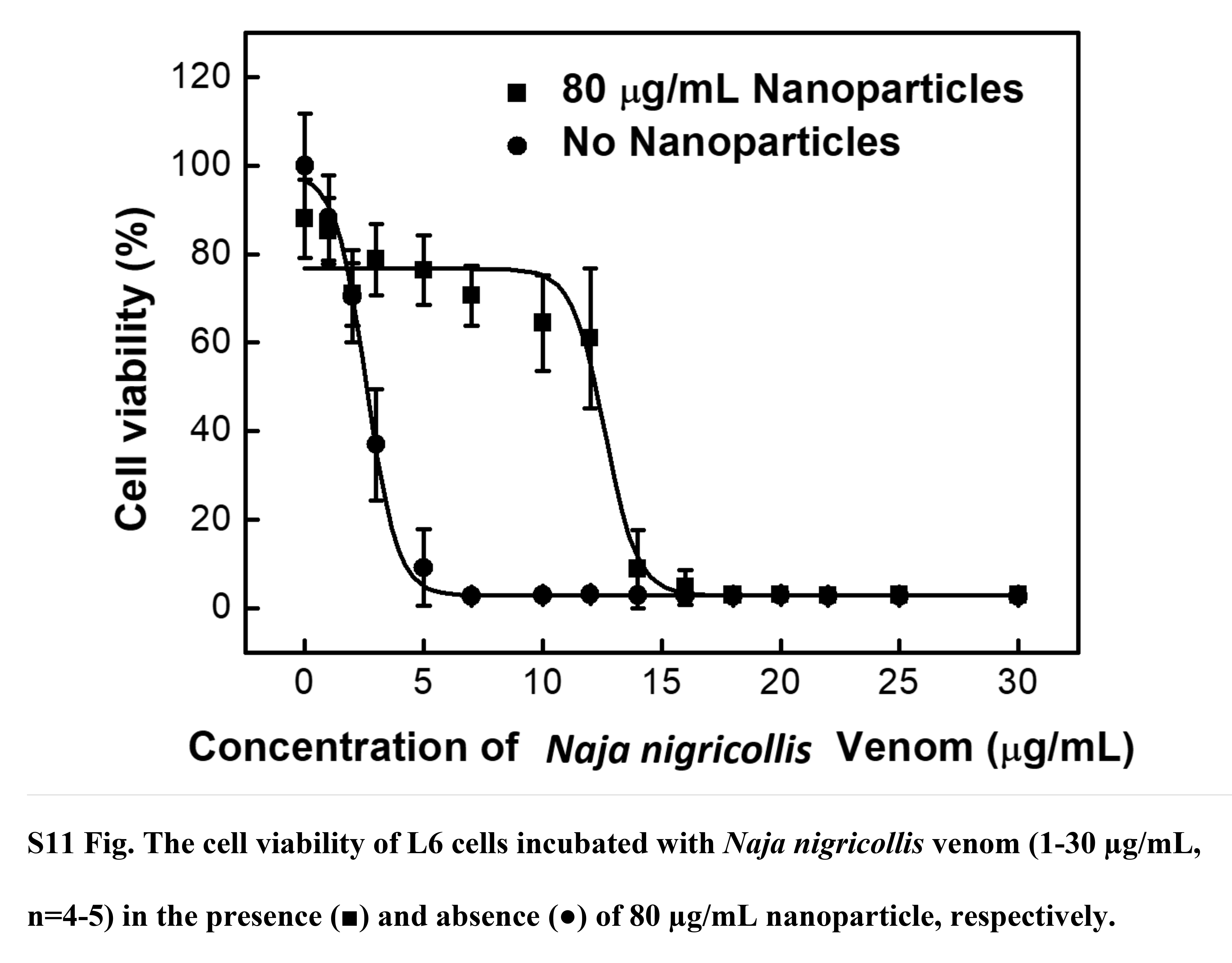

Supplement: S11 Fig — (TIF) [file pntd.0006736.s011.tif]

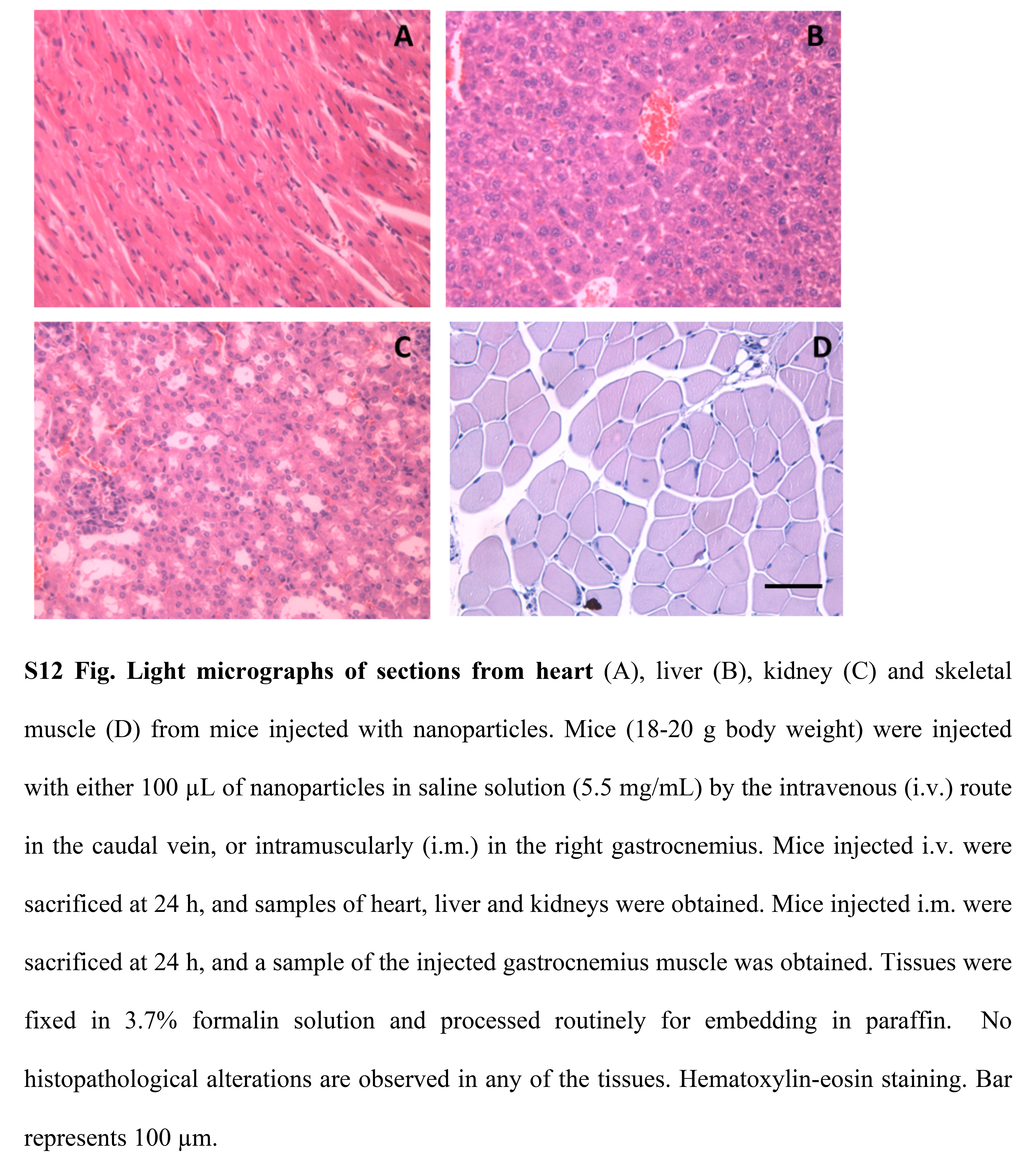

Supplement: S12 Fig — Light micrographs of sections from heart (A), liver (B), kidney (C) and skeletal muscle (D) from mice injected with nanoparticles. Mice (18–20 g body weight) were injected with either 100 μL of nanoparticles in saline solution (5.5 mg/mL) by the intravenous (i.v.) route in the caudal vein, or intramuscularly (i.m.) in the right gastrocnemius. Mice injected i.v. were sacrificed at 24 h, and samples of heart, liver and kidneys were obtained. Mice injected i.m. were sacrificed at 24 h, and a sample of the injected gastrocnemius muscle was obtained. Tissues were fixed in 3.7% formalin solution and processed routinely for embedding in paraffin. No histopathological alterations are observed in any of the tissues. Hematoxylin-eosin staining. Bar represents 100 μm. (TIF) [file pntd.0006736.s012.tif]

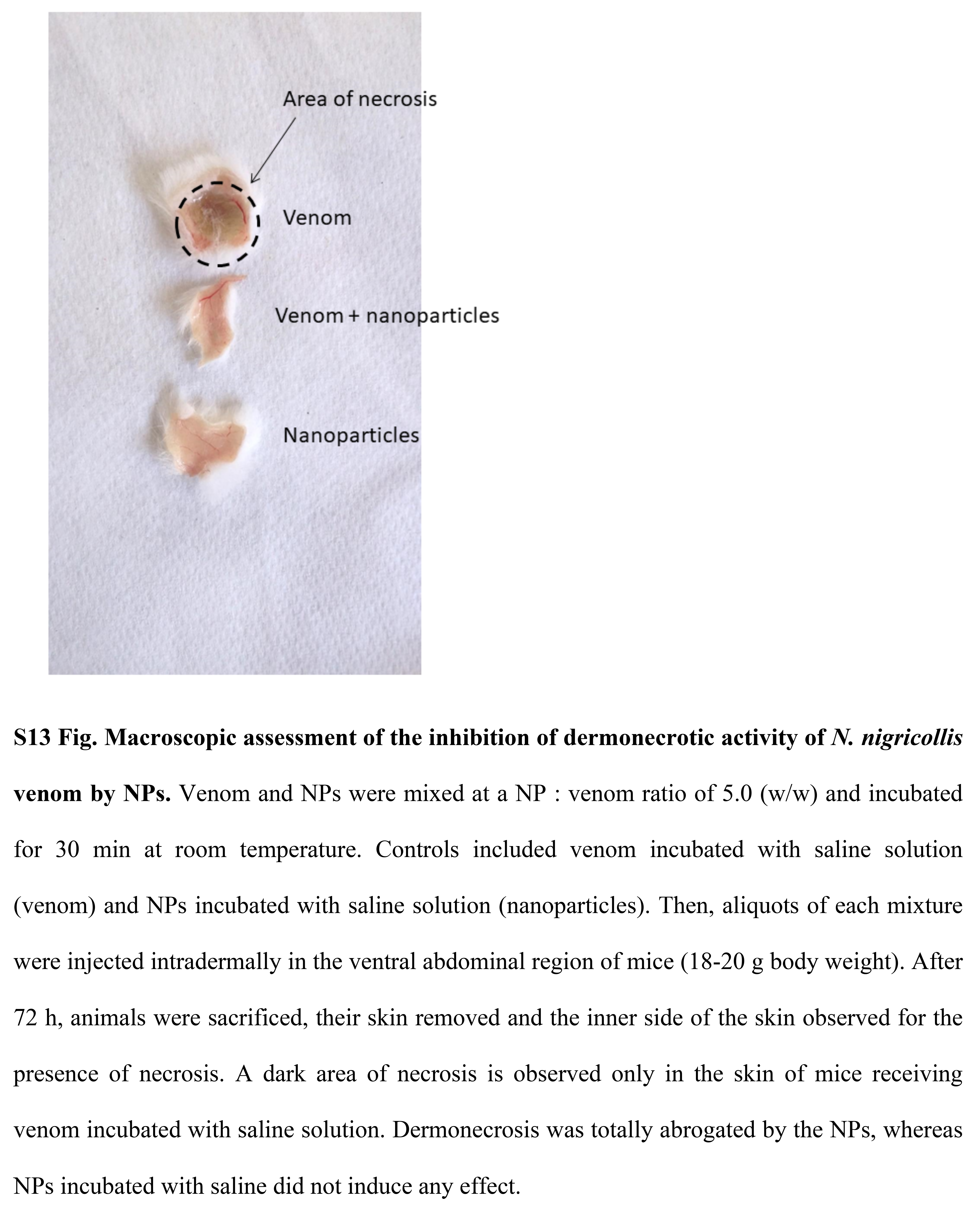

Supplement: S13 Fig — Venom and NPs were mixed at a NP: venom ratio of 5.0 (w/w) and incubated for 30 min at room temperature. Controls included venom incubated with saline solution (venom) and NPs incubated with saline solution (nanoparticles). Then, aliquots of each mixture were injected intradermally in the ventral abdominal region of mice (18–20 g body weight). After 72 h, animals were sacrificed, their skin removed and the inner side of the skin observed for the presence of necrosis. A dark area of necrosis is observed only in the skin of mice receiving venom incubated with saline solution. Dermonecrosis was totally abrogated by the NPs, whereas NPs incubated with saline did not induce any effect. (TIF) [file pntd.0006736.s013.tif]

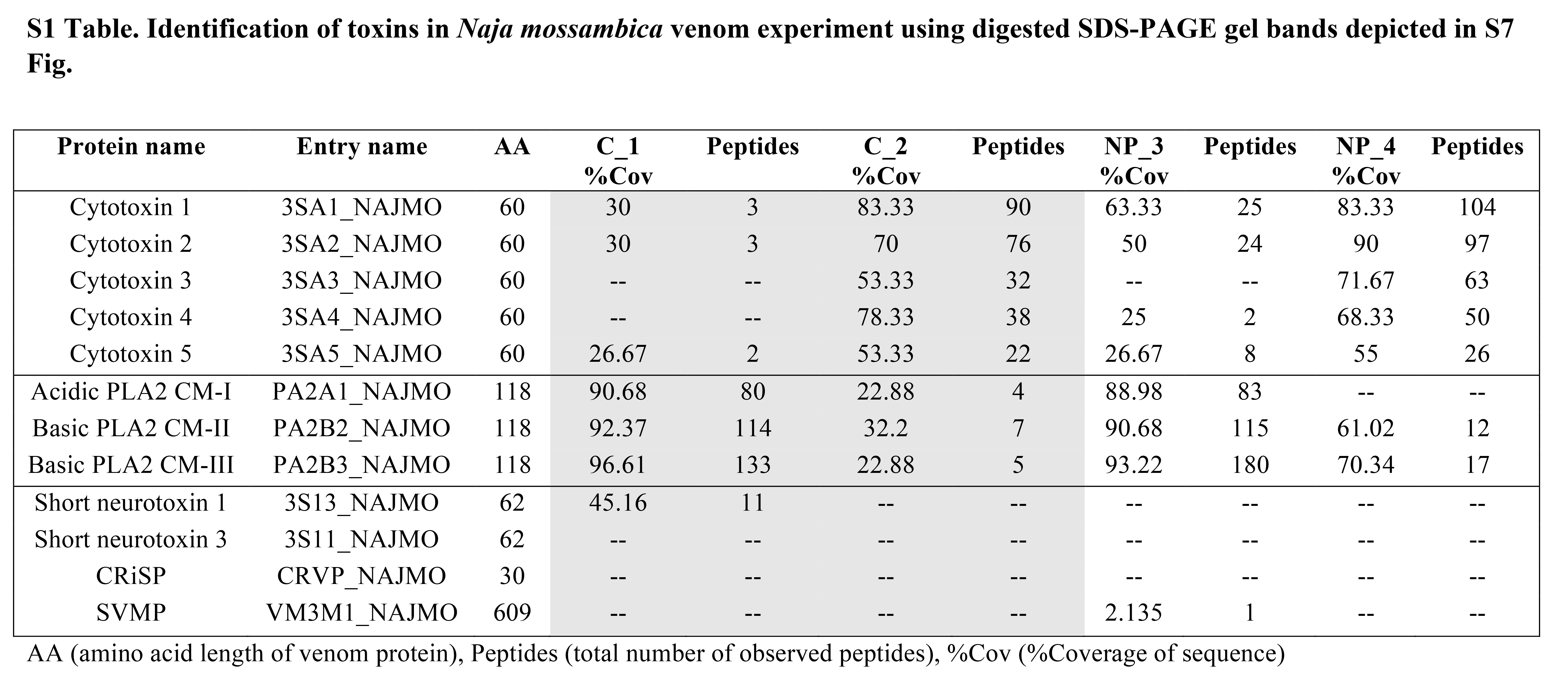

Supplement: S1 Table — (TIF) [file pntd.0006736.s014.tif]

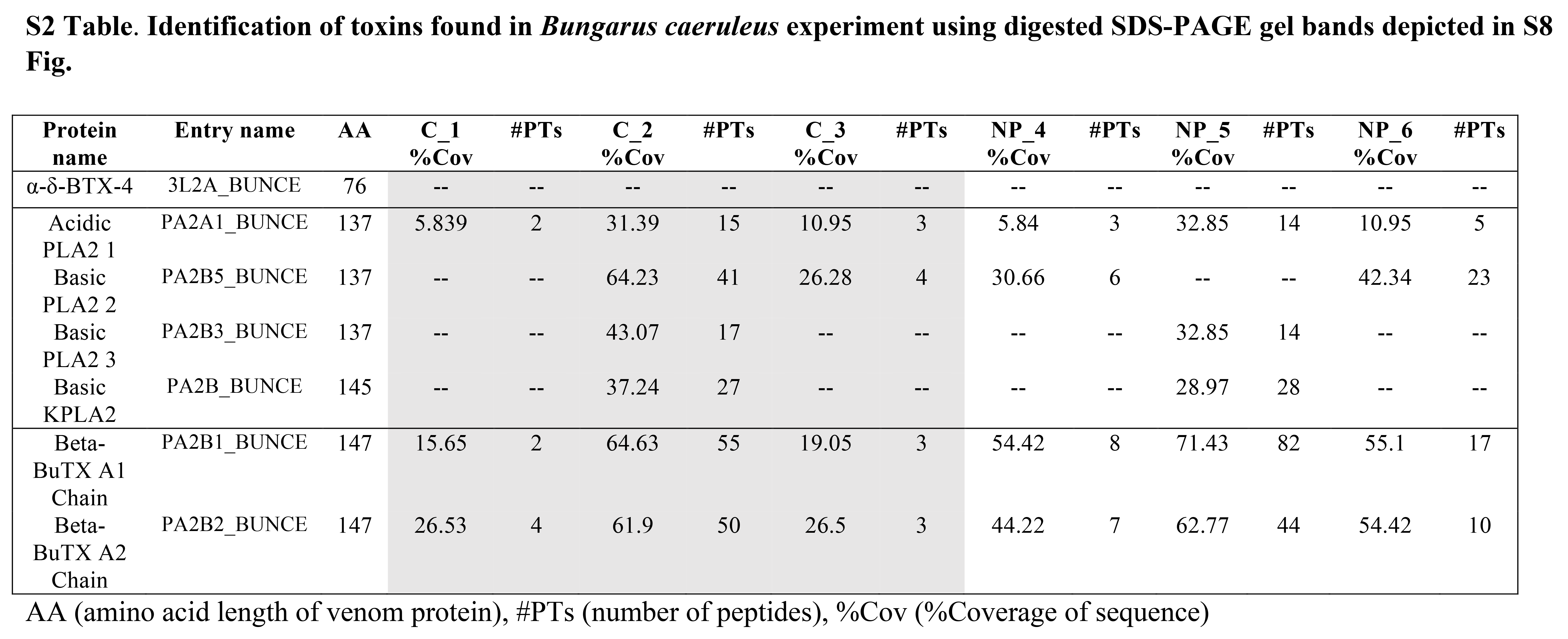

Supplement: S2 Table — (TIF) [file pntd.0006736.s015.tif]

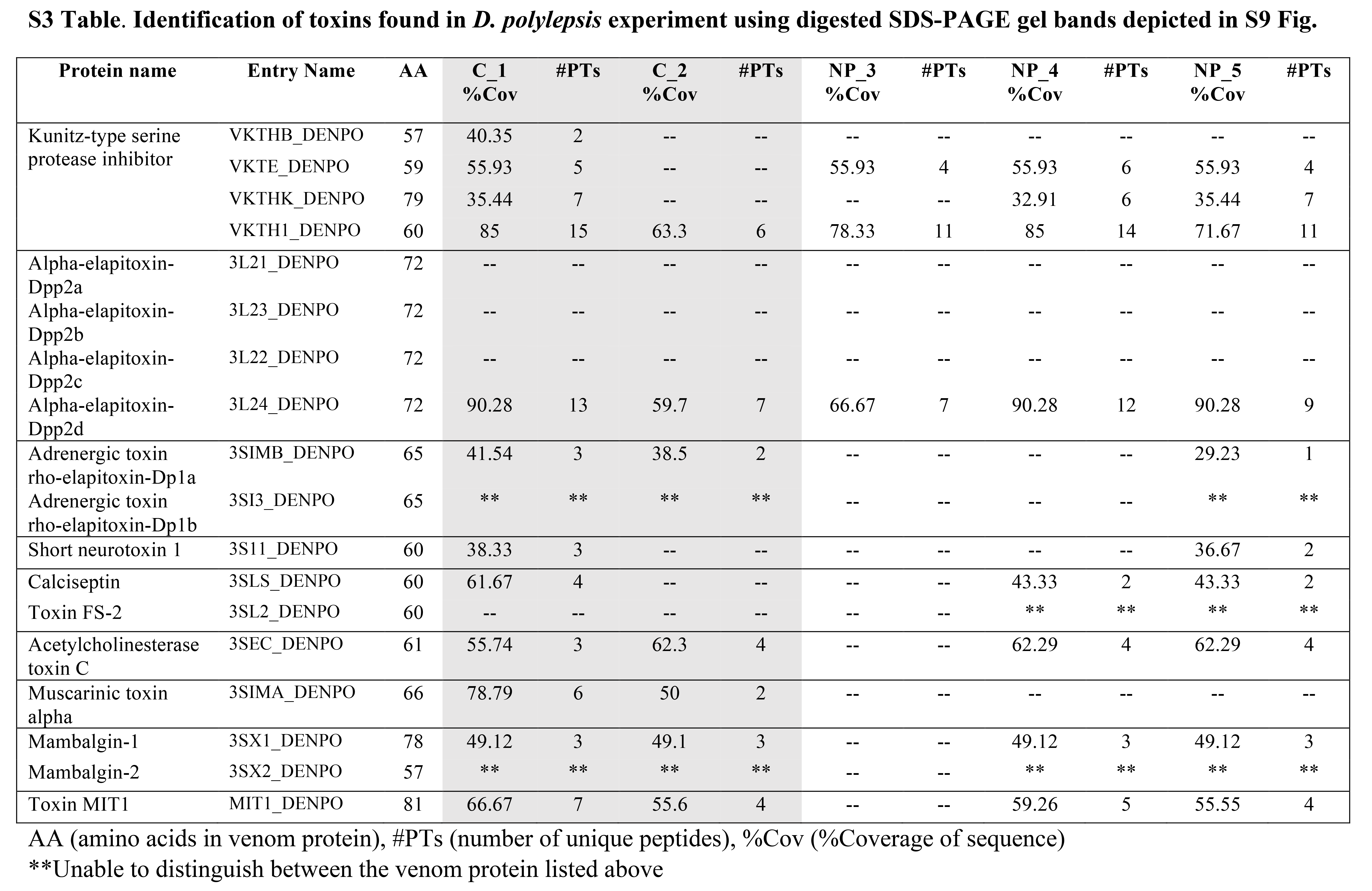

Supplement: S3 Table — (TIF) [file pntd.0006736.s016.tif]

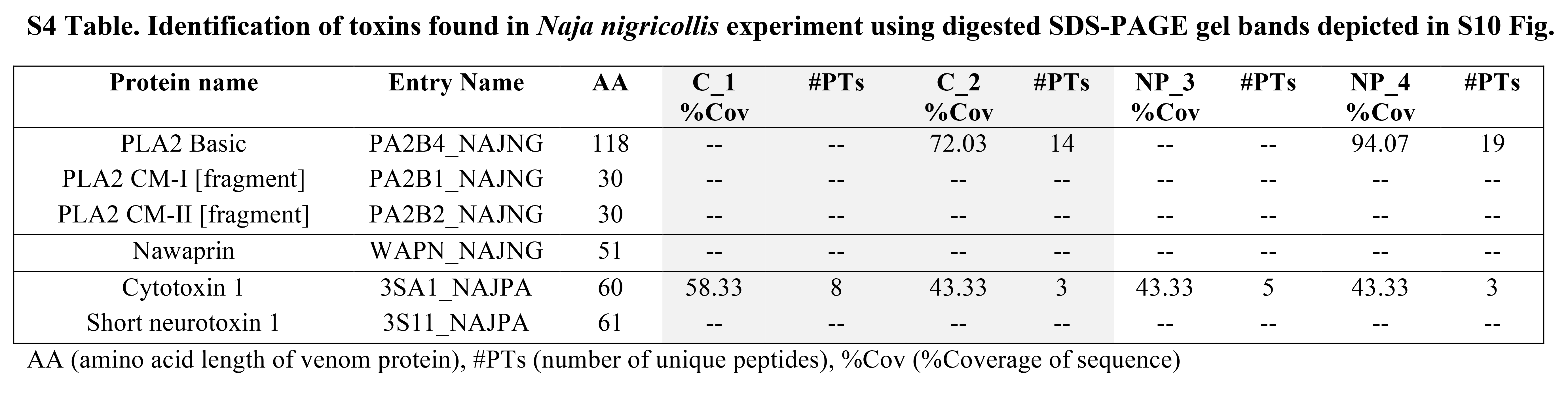

Supplement: S4 Table — (TIF) [file pntd.0006736.s017.tif]
